# Ultrastructural analysis of mitotic *Drosophila* S2 cells identifies distinctive microtubule and intracellular membrane behaviors

**DOI:** 10.1186/s12915-018-0528-1

**Published:** 2018-06-15

**Authors:** Anton Strunov, Lidiya V. Boldyreva, Evgeniya N. Andreyeva, Gera A. Pavlova, Julia V. Popova, Alena V. Razuvaeva, Alina F. Anders, Fioranna Renda, Alexey V. Pindyurin, Maurizio Gatti, Elena Kiseleva

**Affiliations:** 1 Institute of Molecular and Cellular Biology, Siberian Branch of RAS, Novosibirsk, 630090 Russia; 2 Institute of Cytology and Genetics, Siberian Branch of RAS, Novosibirsk, 630090 Russia; 3 Novosibirsk State University, Novosibirsk, 630090 Russia; 4 IBPM CNR and Department of Biology and Biotechnology, Sapienza University of Rome, 00185 Rome, Italy; 5 Present address: Wadsworth Center, New York State Department of Health, Albany, NY 12201 USA

**Keywords:** S2 cells, *Drosophila*, Nuclear membranes, Lamin, Spindle microtubules, Kinetochores

## Abstract

**Background:**

S2 cells are one of the most widely used *Drosophila melanogaster* cell lines. A series of studies has shown that they are particularly suitable for RNAi-based screens aimed at the dissection of cellular pathways, including those controlling cell shape and motility, cell metabolism, and host–pathogen interactions. In addition, RNAi in S2 cells has been successfully used to identify many new mitotic genes that are conserved in the higher eukaryotes, and for the analysis of several aspects of the mitotic process. However, no detailed and complete description of S2 cell mitosis at the ultrastructural level has been done. Here, we provide a detailed characterization of all phases of S2 cell mitosis visualized by transmission electron microscopy (TEM).

**Results:**

We analyzed by TEM a random sample of 144 cells undergoing mitosis, focusing on intracellular membrane and microtubule (MT) behaviors. This unbiased approach provided a comprehensive ultrastructural view of the dividing cells, and allowed us to discover that S2 cells exhibit a previously uncharacterized behavior of intracellular membranes, involving the formation of a quadruple nuclear membrane in early prometaphase and its disassembly during late prometaphase. After nuclear envelope disassembly, the mitotic apparatus becomes encased by a discontinuous network of endoplasmic reticulum membranes, which associate with mitochondria, presumably to prevent their diffusion into the spindle area. We also observed a peculiar metaphase spindle organization. We found that kinetochores with attached k-fibers are almost invariably associated with lateral MT bundles that can be either interpolar bundles or k-fibers connected to a different kinetochore. This spindle organization is likely to favor chromosome alignment at metaphase and subsequent segregation during anaphase.

**Conclusions:**

We discovered several previously unknown features of membrane and MT organization during S2 cell mitosis. The genetic determinants of these mitotic features can now be investigated, for instance by using an RNAi-based approach, which is particularly easy and efficient in S2 cells.

**Electronic supplementary material:**

The online version of this article (10.1186/s12915-018-0528-1) contains supplementary material, which is available to authorized users.

## Background

Schneider 2 cells, commonly called S2 cells, are one of the most widely used *Drosophila melanogaster* cell lines. S2 cells were established from a primary culture of late stage embryos and are likely to be derived from a phagocytic hematopoietic cell lineage [1, 2]. Extensive work carried out in the past 15 years has shown that *Drosophila* S2 cells are an excellent system for the molecular dissection of mitosis using RNA interference (RNAi). There are multiple advantages for using S2 cells in RNAi-based studies of mitosis. First, they have a very favorable cytology for spindle and chromosome visualization in both fixed and living cells [3, 4]. Second, RNAi in S2 cells is very easy because it can be carried out by treating cultures with large double-stranded RNAs (dsRNAs; of 400–800 bp), which are incorporated by the cells and processed into small interfering RNAs (siRNAs) that efficiently degrade the target mRNA [5, 6]. Additionally, simultaneous RNAi against multiple genes can be performed, allowing pathway dissection. Third, the *Drosophila* genome is fully sequenced and well annotated and mitotic genes are highly evolutionarily conserved among metazoans [7, 8], allowing RNAi data on S2 cells to be extrapolated to human cells.

Over the past 15 years, hundreds of papers have been published on exploiting RNAi to study the roles of individual *Drosophila* genes in S2 cell mitosis. RNAi in S2 cells has also been used to analyze the functions of specific groups of genes, such as those encoding for proteins involved in cytokinesis [9], microtubule (MT)-based motor proteins [3], MT-binding proteins [10], protein kinases [11], and protein phosphatases [12]. In addition, S2 cells have been used to perform RNAi-based genome-wide screens to identify genes involved in different aspects of cell division [4, 13] or in specific aspects of the process, such as cytokinesis [14], centrosome assembly and behavior [15], or centrosome-independent spindle assembly [16]. Collectively, these studies have identified many new mitotic genes and provided fundamental insight into the molecular mechanisms of cell division.

Despite the enormous work carried out on S2 cells mitosis, no detailed and complete description of the process at the ultrastructural level has been done. Here, we analyzed S2 cell mitosis by transmission electron microscopy (TEM). We examined sections from a random sample of 144 cells undergoing mitotic division. This unbiased approach allowed us to discover that S2 cells exhibit peculiar intracellular membrane behaviors, and to subdivide prometaphase into four stages. Our observations also indicate that in late S2 prometaphase and metaphase, the spindle MTs are mostly organized in bundles, which are either end-on attached to the kinetochores (k-fibers) or run from pole to pole (interpolar MTs). In addition, kinetochores with attached k-fibers almost invariably exhibit lateral associations, with either k-fibers connected to different kinetochores or interpolar MT bundles, forming multi-bundle MT assemblies that are likely to aid chromosome alignment at the metaphase and poleward movement in the anaphase.

## Results and discussion

To describe *Drosophila* mitosis at the ultrastructural level, actively proliferating *Drosophila* S2 cells were pelleted, fixed, embedded in resin, and sectioned. Because cells within a pellet are randomly oriented, this procedure yielded sections along different planes of the mitotic apparatus. We examined only cells cut parallelly or slightly obliquely with respect to the spindle axis (henceforth, longitudinal sections), or cut through the mitotic chromosomes perpendicularly to the spindle axis (henceforth, transverse sections). Oblique cuts showing only part of the mitotic cell were discarded. For 67% of the cells, we analyzed single ultrathin sections (of approximately 70 nm). For the remaining cells, we performed serial sectioning and examined up to 15 sections per cell, mainly to obtain precise information on the intracellular membranes and kinetochore–MT relationships. To measure cell structures, we analyzed longitudinal and transverse 70 nm sections. For serial sections, we considered only the central section of the series.

Most of the cells in the pellets were in interphase, characterized by a rather homogeneous nucleoplasm and a prominent nucleolus. In the sections of these cells, the nucleus was usually circular and surrounded by a double membrane (Additional file 1: Figure S1a). We also observed cells that, in addition to the nucleolus, displayed several dense aggregations of chromatin and had the nucleus surrounded by an undulated double membrane. These cells might be in prophase or in a pre-prophase stage (Additional file 1: Figure S1b, c).

We focused on 144 cells undergoing mitotic division: 71 in prometaphase (49%), 37 in metaphase (26%), 21 in anaphase (15%), and 15 in telophase (10%). In fixed S2 cells stained for tubulin and DNA and examined under a light microscope (*n* = 369), we found 40% prometaphases, 20% metaphases, 11% anaphases, and 29% telophases. Clearly, there is an excess of telophases compared to the TEM preparations. However, this is easily explainable, considering the difficulty in obtaining TEM sections with complete telophase figures including both presumptive daughter cells versus those that contain a single daughter cell; the latter were excluded from our analysis. If we do not consider telophases, the relative frequencies of prometaphases, metaphases and anaphases observed in TEM preparations (55%, 29%, and 16%, respectively) and in light microscopy specimens (56%, 28%, and 16%, respectively) are almost identical. Thus, at least for prometaphases, metaphases, and anaphases, the TEM sample of mitotic cells we examined is representative of a population of actively proliferating S2 cells. We focused on intracellular membrane and MT behavior. The centriole ultrastructure has been described previously [17–19].

*Drosophila* mitosis is semi-closed, since prometaphase is not preceded by nuclear envelope breakdown (NEBD) but begins when nuclear envelope fenestrations at the cell poles allow penetration of astral MTs into the nucleus [20, 21]. Consistent with this, we found that approximately one-half of prometaphases were surrounded by nuclear membranes. These cells were real prometaphases, since they exhibited both compact chromatin patches and MTs within the nucleoplasm. According to their morphology, we subdivided prometaphases into four classes: PM1 through PM4. To determine the temporal sequence of these classes, we considered several parameters: the chromatin compaction, the MT arrangement, the structure of kinetochores, and the intracellular membrane organization. In addition, we considered nuclear lamina behavior, which is described below. Metaphases were recognized based on the same parameters and, in many cases, on the examination of serial sections, and were included in a single class. Anaphases were subdivided into early and mid/late, and telophases were subdivided into early/mid and late.

### Prometaphase

**Fig. 1:**
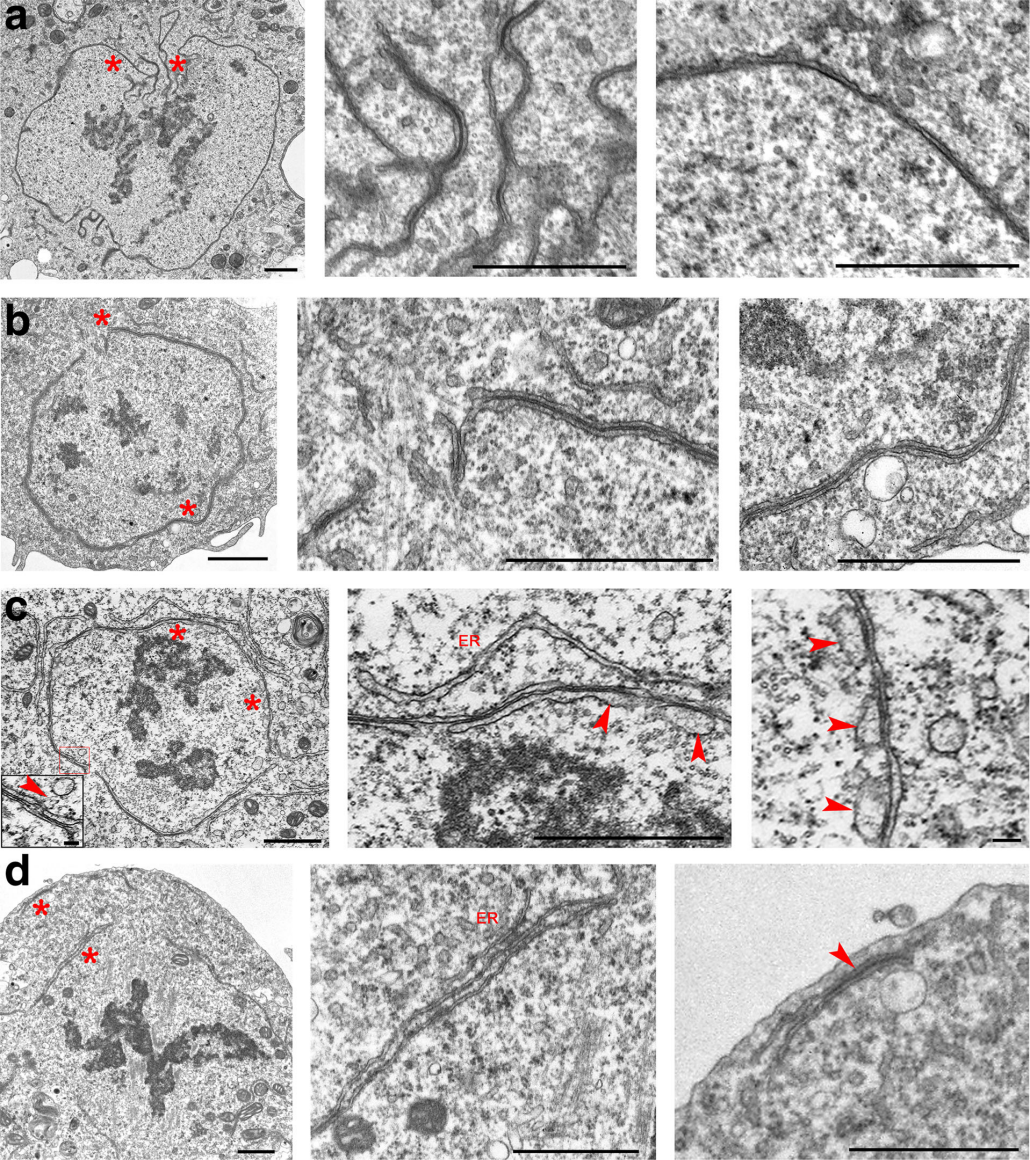
Prometaphase stages of S2 cells. **a** PM1 stage. Note the formation of QNM in the area of the nuclear fenestration and the presence of a normal DNM along most of the nuclear envelope. **b** PM2 stage. Note that almost the entire nuclear envelope is composed by QNM. **c** PM3 stage. Note the disassembly of the inner membrane of the QNM through vesiculation. Inset: Vesicles are indicated by arrowheads. **d** PM4 stage. Note the almost complete disassembly of the QNM (remnants of the QNM are observed at the cell poles, arrowhead) and the appearance of ER membrane stacks. Asterisks indicate the cell regions shown at higher magnification in the central and rightmost images. Scale bars: 1 μm, except rightmost image of **c**, 0.1 μm. DNM double nuclear membrane, ER endoplasmic reticulum, QNM quadruple nuclear membrane

**Fig. 2:**
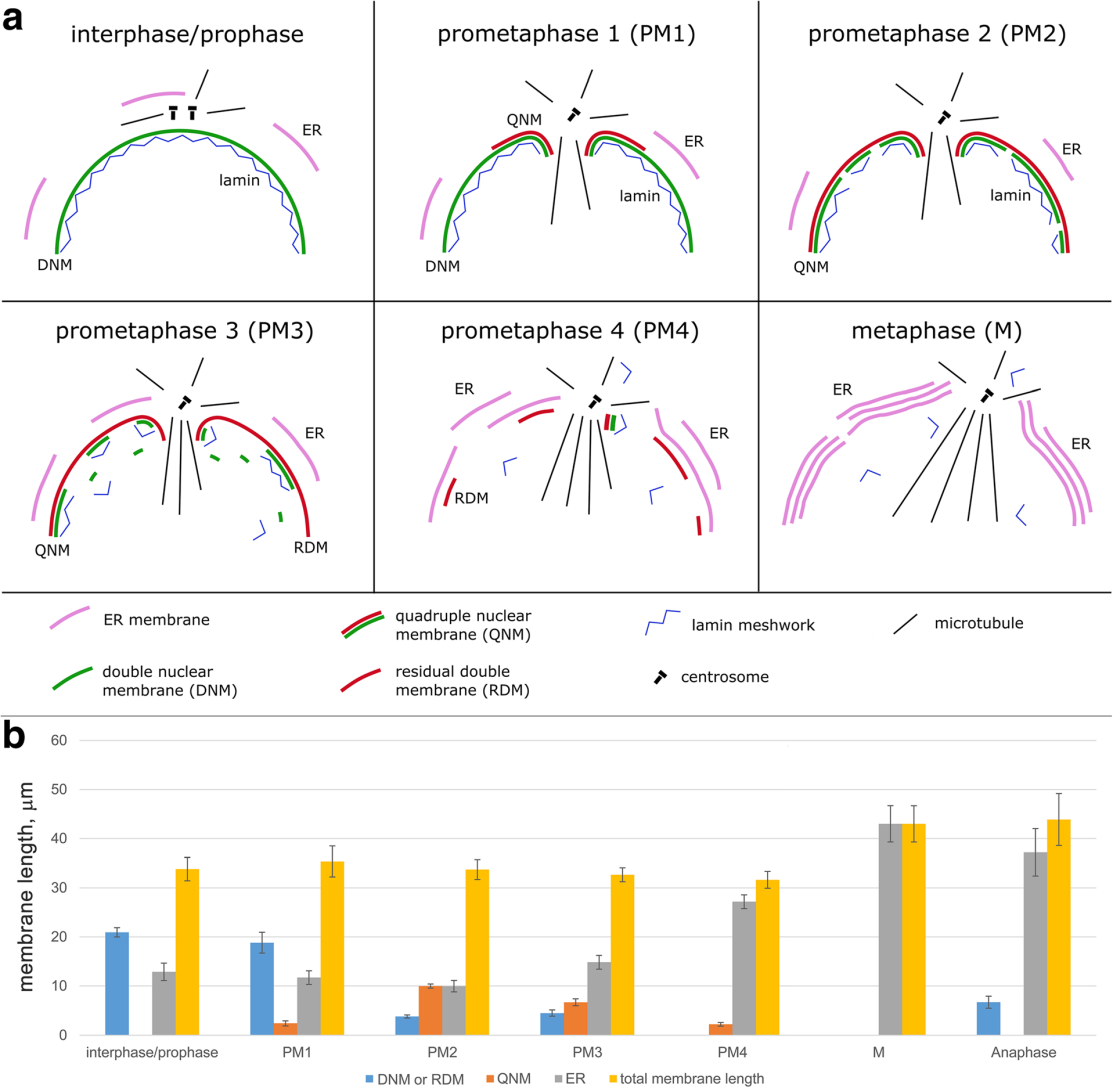
Schematic representation of intracellular membrane behavior during mitosis of S2 cells. **a** Membrane remodeling during prometaphase and metaphase. In PM1, a QNM begins to form in the proximity of the nuclear fenestration area, and during PM2, it extends along the entire nuclear envelope. During late PM2 and PM3, the inner double membrane of the QNM becomes swollen and progressively disassembles through a vesiculation process, while a RDM persists around the nucleus. In PM4, only small patches of QNM are observed at the cell poles and the RDM disassembles. In metaphase, multilayer stacks of ER membranes form alongside the spindle. **b** Membrane length measured in 70 nm sections of interphase and mitotic cells: interphase/prophase (seven cells), PM1 (nine cells), PM2 (seven cells), PM3 (15 cells), PM4 (13 cells), metaphase (12 cells), and anaphase (seven cells). The DNM is present in interphase and prophase cells, in PM1 cells, and around some regions of anaphase chromosomes. In PM3 cells, the DNM is no longer present and the non-QNM portions of the nuclear envelope are made of RDM. To calculate the total membrane length, we counted the QNM twice. Note that the total membrane length stays mainly constant from prophase through to late prometaphase, and increases in metaphase and anaphase. Error bars are for the standard error of the mean. DNM double nuclear membrane, ER endoplasmic reticulum, QNM quadruple nuclear membrane, RDM residual double membrane

We assigned 12 prometaphases to the PM1 class. To define the characteristic features of this class, we examined serial transverse sections through the chromosomes of one cell, and at least one longitudinal or slightly oblique section with respect to the spindle axis for each of the 11 other cells. For two of these 11 cells, we generated serial sections (an example of these serial sections is shown in Additional file 2: Figure S2a). Cells in the PM1 class are characterized by the presence of a continuous membrane envelope (except at nuclear fenestrations) around the nucleus, mainly comprising a typical double nuclear membrane (DNM) but also patches of a quadruple membrane (QNM) formed by two closely apposed double membranes connected by a layer of electron-dense material of unknown nature. The outer and inner membranes of the QNM and the central electron-dense material span 25.5 ± 0.8, 24.9 ± 0.6, and 18.5 ± 0.8 nm, respectively, with a total width of 68.9 ± 1.8 nm. In all cases, the QNM did not represent more than 18% of the total nuclear envelope length. In cells where fenestrations in the nuclear envelope were visible (in six of the 11 longitudinally cut cells), the QNM was always located in these regions and exhibited ribosomes on both its outer and inner surfaces (Figs. 1a and 2a, and Additional file 2: Figure S2a). For the remaining five cells, patches of QNM were clearly present but could not be associated with nuclear fenestrations, as these membrane openings could not be seen due to the sectioning plane. In 10 of the 12 PM1 cells, we observed patches of endoplasmic reticulum (ER) membranes all over the cytoplasm. Their average length per cell section was 11.7 ± 1.6 μm, while the length of the membranes forming the nuclear envelope (DNM plus 2 × QNM) was 23.7 ± 2.8 μm (Fig. 2b). An analysis of MTs in the transversely cut cell indicated that, at this stage of mitosis, MTs are rather sparse and are not organized in bundles as in later stages (Additional file 3: Figure S3a). In addition, in two of the 11 longitudinally cut cells, we were able to see immature kinetochores, namely kinetochores with an oblong shape that do not appear to interact with MTs in an end-on fashion (Additional file 4: Figure S4).

The prometaphase cells of the PM2 and PM3 classes show similar features and a continuum of morphological variations. They both exhibit a higher chromatin compaction compared to PM1 cells and have a mostly continuous nuclear envelope that contains regions of QNM varying in length from 20% to 80% of the nuclear envelope. In some of the regions made of QNM, the inner membrane showed unevenly swollen traits and some discontinuities, suggesting that it is undergoing some form of decay (Figs. 1b, c, and 2a, and Additional file 2: Figure S2b, c). In other nuclear envelope regions of variable length, the inner membrane of the QNM had completely disappeared (Figs. 1b, c, and 2a, and Additional file 2: Figure S2b, c), leaving a residual double membrane (RDM). We observed ribosomes associated with both the outer and inner surfaces of the QNM as well as with both surfaces of the RDM (Fig. 1b and Additional file 2: Figure S2b, c).

We assigned 17 cells to the PM2 class based on the fact that their nuclear envelopes are formed by at least 60% of QNM. The inner membrane swelling was limited or absent in these cells, providing a criterion for distinguishing them from the PM3 stages, in which the inner membrane is often deformed. The PM2 class includes one cell represented by serial transverse sections through the chromosomes, and 16 longitudinally sectioned cells, three of which are represented by serial sections. In 13 of these 17 PM2 cells, we observed patches of ER membranes, whose average length per cell section was 10.0 ± 1.2 μm, while the length of the membranes forming the nuclear envelope (2 × QNM plus RDM) was 23.8 ± 1.3 μm (Fig. 2b). An analysis of the transverse sections indicated that also in this stage of mitosis, MTs are rather sparse, although some loose MT bundles were apparent (Additional file 3: Figure S3b). In four of the 16 longitudinally cut cells, we were able to see immature kinetochores (Additional file 4: Figure S4).

We assigned to the PM3 stage 18 cells with nuclear envelopes containing less than 60% of QNM. This class includes two cells cut transversely through the chromosomes, one of which was serially sectioned, and 16 longitudinally cut cells, nine of which are represented by serial sections. In these PM3 cells, the inner membrane of the QNM was usually more swollen and more discontinuous than in PM2 cells. In several regions of the PM3 cells, the swollen membrane was ~80 nm wide, while in the few regions of PM2 cells showing inner membrane deformation, the swollen membrane was ~40 nm wide (in non-swollen regions, the inner membrane was ~25 nm wide). These observations suggest that the inner component of the QNM is undergoing a progressive degradation process through vesiculation (Figs. 1c and 2a, and Additional file 2: Figure S2c). Of the 18 cells in the PM3 class, 16 showed layers of ER membranes surrounding more or less extensive regions of the nuclear envelope (formed by QNM and RDM). In these 16 cells, the average length of the ER membranes per cell section was 14.8 ± 1.4 μm, while the length of the membranes forming the nuclear envelope (2 × QNM plus RDM) was 17.8 ± 1.8 μm (Fig. 2b). When the ER membrane layers were sufficiently long, their curvatures tended to conform to that of the nuclear envelope (Additional file 2: Figure S2c, and Additional file 3: Figure S3c). However, in contrast with the typical QNM appearance, ER membranes rarely showed close apposition to either the QNM or the RDM. We observed only a few cells where this type of adhesion appeared to occur in a limited portion of the nuclear envelope (Additional file 5: Figure S5). An analysis of MTs in transverse sections indicated that in this stage of mitosis there is a diminution of isolated MTs compared to the PM2 class (Additional file 3: Figure S3c). Some MT bundles were visible, but they were fewer than the bundles observed in the following stage of prometaphase (PM4) and in metaphase. In 11 of the 18 cells examined, we were able to see immature kinetochores (Additional file 4: Figure S4).

We assigned 24 cells to the PM4 class. Three were transversely sectioned (one serially) and 21 were longitudinally sectioned (six serially). These cells are characterized by compact chromatin and the disappearance of almost all the QNM and RDM. However, most spindles of the PM4 cells (19 out of 24) were partly enveloped by one or more irregular layers of ER membranes comprising from one to three layers running alongside with little or no overlap (Fig. 1d). In the 19 cells with ER membranes, the average length of these membranes was 27.2 ± 1.4 μm per cell section, while the length of the remaining 2× QNM plus RDM was 5.8 ± 1.6 μm (Fig. 2b). An analysis of MTs in transverse sections indicated that in PM4 cells, most MTs are organized in bundles containing from four to 45 MTs (Additional file 3: Figure S3d). In addition, in a substantial fraction of longitudinal sections (eight out of 21), we were able to see mature kinetochores, most of them showing end-on MT attachment (Additional file 4: Figure S4).

### Metaphase

**Fig. 3:**
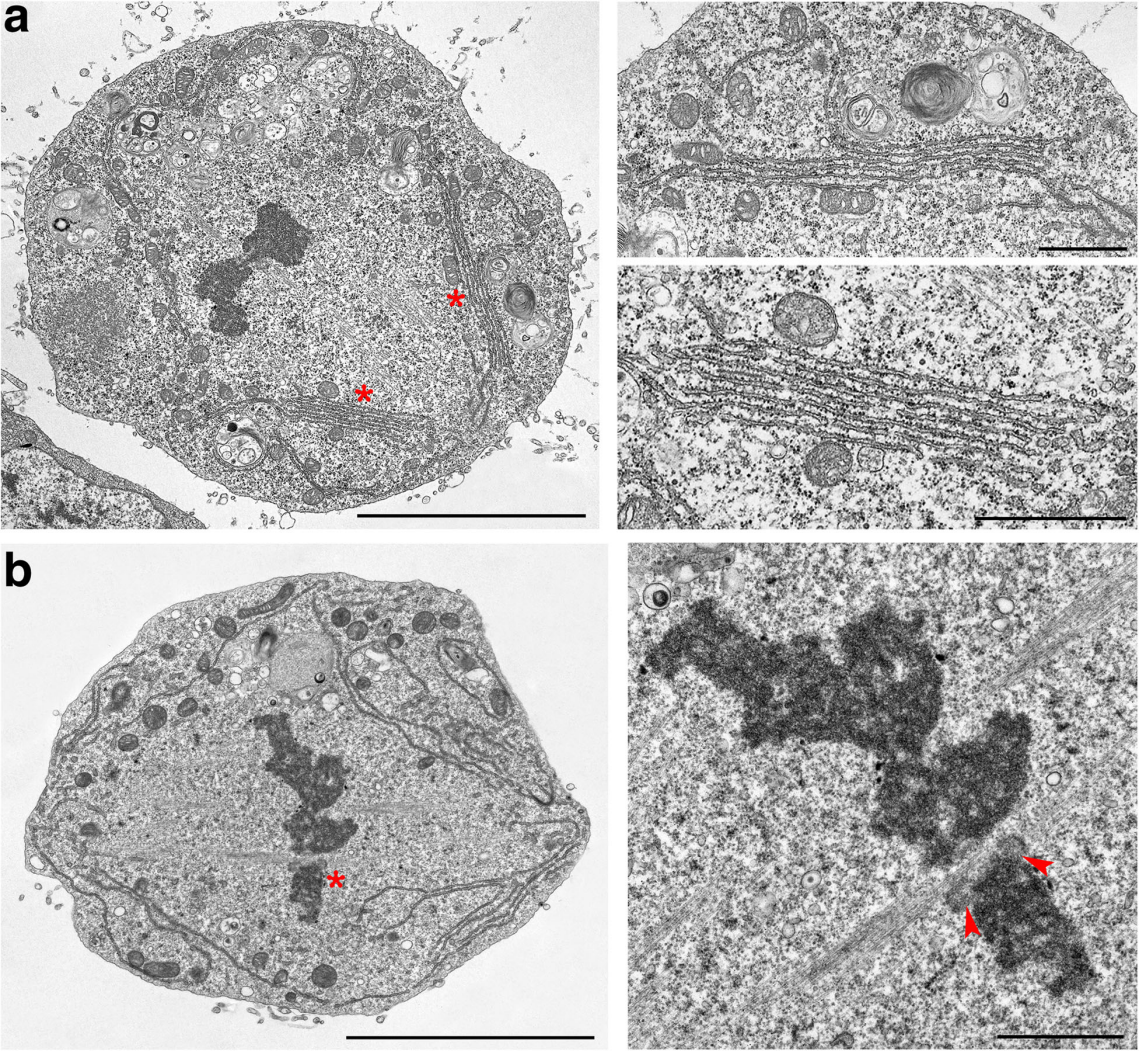
Examples of metaphase S2 cells. Note (**a**) the stacks of parallel endoplasmic reticulum membranes and (**b**) the kinetochores with k-fibers (arrowheads). Asterisks indicate the cell regions shown at higher magnification in the images on the right. Scale bars: left images, 5 μm; right images, 1 μm

The 37 cells assigned to this class include eight transversely sectioned through the chromosomes (four of which are represented by serial sections) and 29 longitudinally cut cells (11 of which were serially sectioned). These cells are characterized by the alignment of highly compacted chromosomes at the cell equator and by the presence of robust MT bundles often ending at the kinetochores (Fig. 3 and Additional file 6: Figure S6). In 26 of the 29 longitudinal sections examined, the spindles and chromosomes were surrounded by one or more discontinuous layers of ER membranes located near the cell poles, at the cell equator, or at both the poles and the equator. Most of these metaphases showed two or three parallel ER membrane layers but some of them displayed from four to seven membrane layers (Figs. 2a and 3a, and Additional file 6: Figure S6a). The cell shown in Fig. 3a exhibits seven parallel layers of ER membranes. In sections showing multiple ER membrane layers, these membranes run alongside the spindle with little or no overlap, and never formed a quadruple membrane comparable to that observed in prometaphase cells. Examination of multiple sections of the same cell showed that the ER membrane layers that surround the metaphase spindles are organized in discontinuous and irregular stacks, which contain a variable number of membranes (see Additional file 2: Figure S2d as an example). Thus, a metaphase spindle is not isolated by a “spindle envelope” but has many regions that are contiguous to the mitotic cytoplasm. In the 26 cells showing ER membranes, the average membrane length per section was 43.0 ± 3.7 μm (Fig. 2b).

Examination of the transverse sections showed that metaphase MTs are mainly organized in bundles. Some of these bundles end directly on the kinetochores while others appear to run alongside the kinetochores (Additional file 6: Figure S6).

### Anaphase

**Fig. 4:**
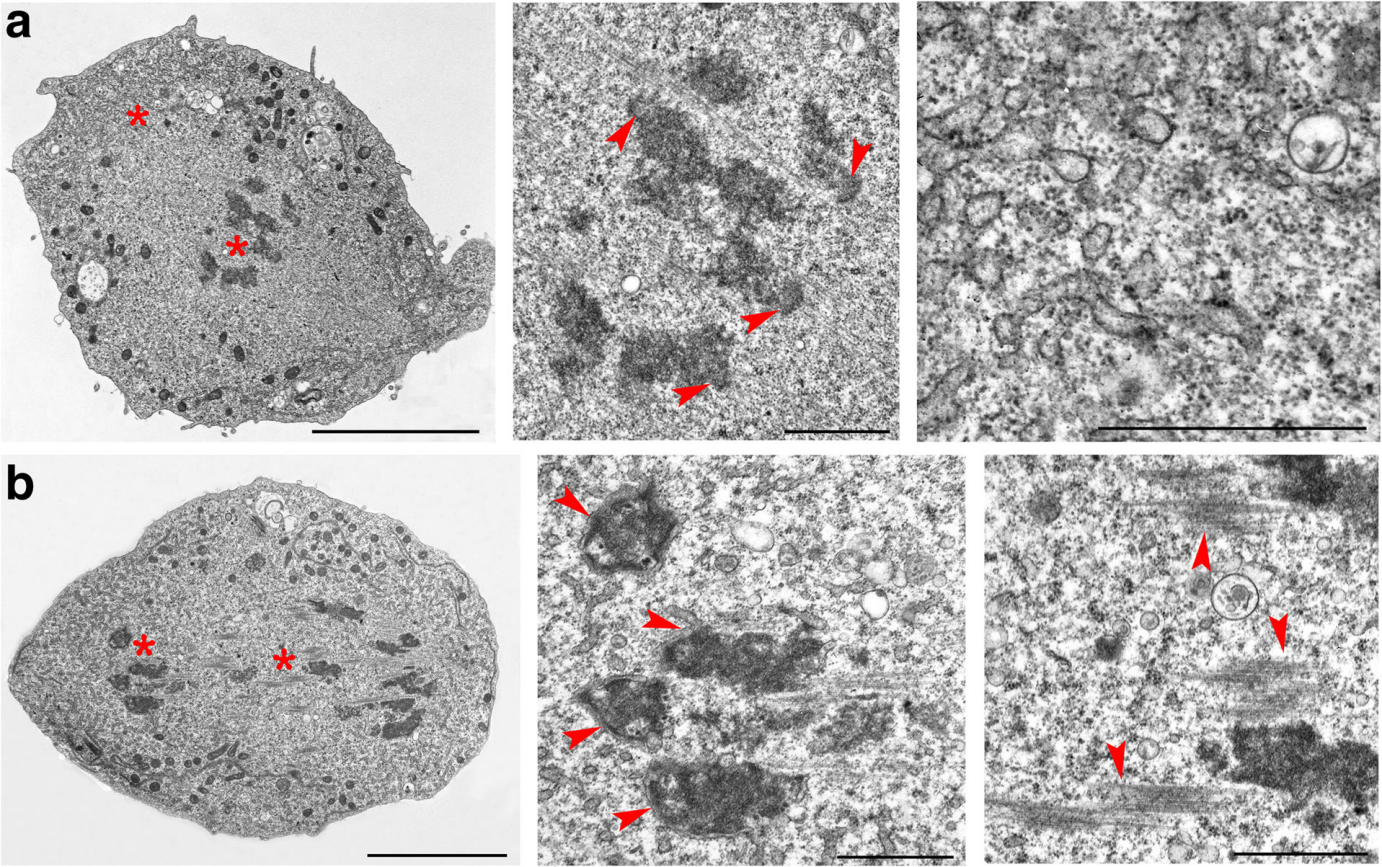
Examples of anaphase S2 cells. **a** Early anaphase cell showing initial separation of the sister chromatids. Note the kinetochores of the separating sister chromatids (arrowheads) and the vesicles at the spindle poles. **b** Mid/late anaphase. Note the formation of a double membrane around the chromosomes (arrowheads) and the MT bundles in the center of the cell. Asterisks indicate the cell regions shown at higher magnification in the central and rightmost images. Scale bars: leftmost image, 5 μm, central and rightmost images, 1 μm

Only longitudinal or slightly oblique sections of anaphase cells were examined. We sectioned 12 early and nine mid/late anaphase cells and made serial sections of seven and five of these cells, respectively. We classified as early anaphases those cells in which the sister chromatids are separated and have started moving towards the poles, although the cells still have an oval shape comparable to that of metaphase cells (Fig. 4a and Additional file 7: Figure S7). The MTs of early anaphase cells were still arranged in bundles. In six of the 12 early anaphase cells examined, we observed clear kinetochore structures (Fig. 4a).

In the mid/late anaphase cells, the two sets of separating chromatids have reached the spindle poles or are located very close to them, and the cells exhibit an elongated shape compared to metaphase cells (Fig. 4b and Additional file 7: Figure S7). In eight of the nine mid/late anaphase cells, we observed large but discontinuous patches of double membrane surrounding the chromosomes. In contrast, only four of the 12 early anaphase cells examined showed very small patches of chromosome-associated membranes (Fig. 4 and Additional file 7: Figure S7). Kinetochores and MT bundles connecting the chromosomes with the spindle poles were never observed in mid/late anaphase cells. However, longitudinal sections of these cells showed bundles of MTs traversing the sets of migrating chromosomes and/or placed between these sets in the center of the cell (Fig. 4b and Additional file 7: Figure S7). The latter MT bundles will probably give rise to the telophase central spindle.

In both early and mid/late anaphase cells, the ER membranes were mostly confined in the polar regions of the cell but also formed single layers alongside the central part of the spindle. These ER membranes were shorter and often undulated compared to those seen in metaphase cells (Fig. 4 and Additional file 7: Figure S7). We measured the ER membranes also in sections of anaphase cells (early and mid/late) and found that their average length was 37.2 ± 4.8 μm per section (Fig. 2b). In mid/late anaphase cells, the average length of DNM forming around the chromosomes was 6.7 ± 1.2 μm (Fig. 2b). The polar regions of anaphase cells also displayed many membrane vesicles (Fig. 4 and Additional file 7: Figure S7), which are likely to be intermediates in the Golgi reassembly process. In S2 cells, like in mammalian cells, the Golgi apparatus disassembles upon entry of the cell into mitosis, giving rise to a large number of fragments, which disperse into the cytoplasm. These fragments progressively reaggregate during anaphase and telophase to eventually reassemble the Golgi stacks in the daughter cells [22–24].

### Telophase

**Fig. 5:**
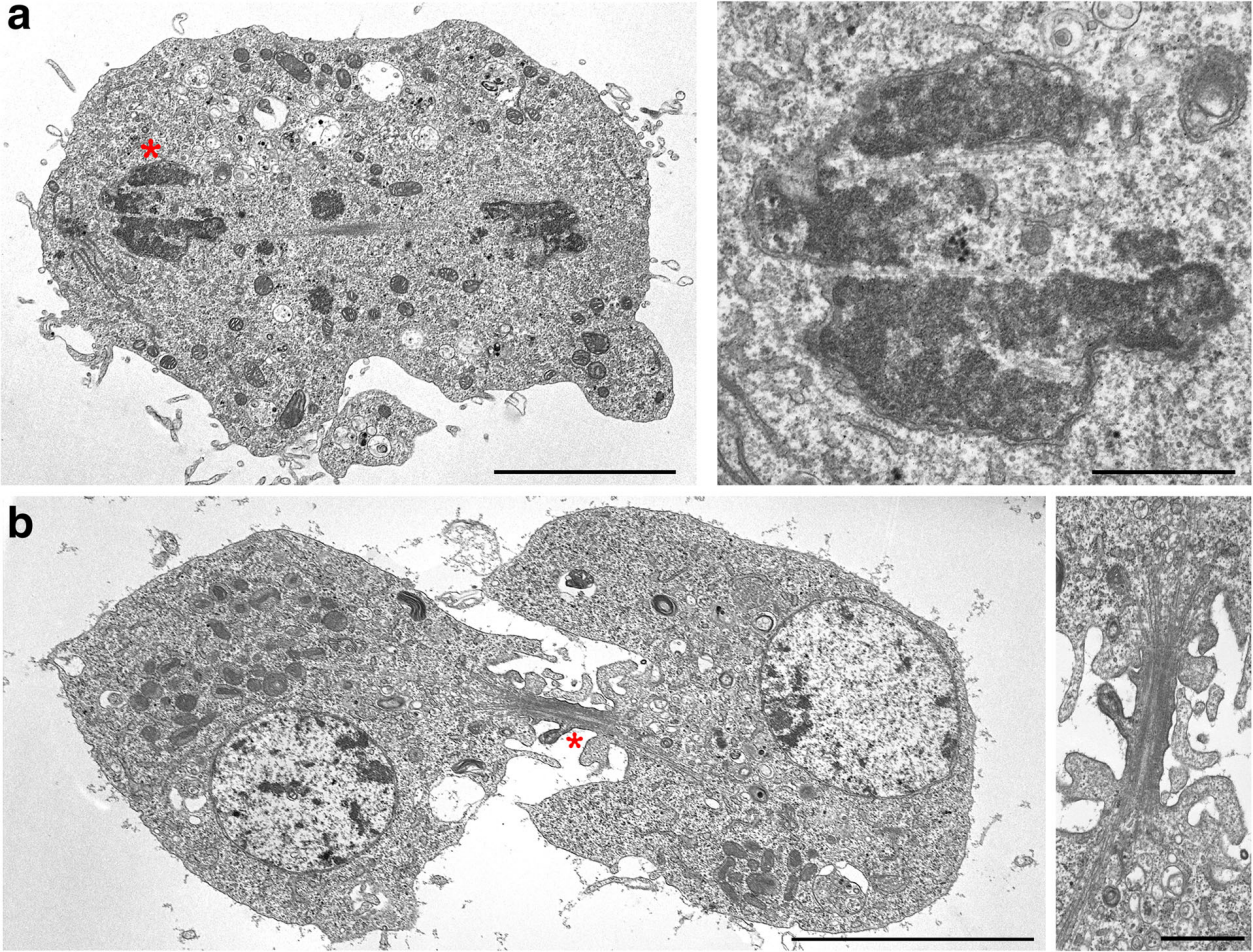
Examples of telophase S2 cells. **a** Early telophase with the chromosomes almost completely surrounded by a double membrane. Note the bulges protruding from both the equatorial region and the cell pole. **b** Late telophase with fully reformed nuclei. Note the multiple protrusions emanating from the intercellular bridge. Asterisks indicate the cell regions shown at higher magnifications in the right panels. Scale bars: left images, 5 μm; right images, 1 μm

Like anaphases, for telophases we examined only longitudinal or slightly oblique sections containing both presumptive daughter cells. We sectioned seven early/mid telophase cells and eight late telophase cells. We had three serial section sets for each of these stages. Early/mid telophase figures were characterized by the decondensation of the chromosomes, which are almost completely surrounded by a DNM. These cells, in addition to an initial cleavage furrow, showed deformations of the surface with frequent bulges protruding from both the equatorial region and the cell poles (Fig. 5a and Additional file 8: Figure S8b). These cell protrusions were not observed in prometaphase, metaphase and anaphase cells. Early/mid telophase cells also exhibited separate MT bundles between the cell poles, in the region where the central spindle is forming (Additional file 8: Figure S8a).

Late telophase cells are characterized by fully reformed nuclei containing decondensed chromatin surrounded by a continuous DNM. The two presumptive daughter cells are connected through an intercellular bridge that contains bundled MTs. In some cells, the plus ends of the MT bundles coming from the opposite daughter cells were clearly overlapping at the center of the intercellular bridge (Additional file 8: Figure S8c, d). One interesting feature of late telophase cells was the presence of many protrusions emanating from the cell surface. Some of these protuberances were rather large but others were small and often exhibited a tube-like appearance. The small protrusions were mostly emanating from the intercellular bridge (Fig. 5b and Additional file 8: Figure S8c, d). Large membrane bulges have been also observed with a light microscope during telophase of normal S2 cells fixed with formaldehyde/phosphate-buffered saline (PBS) and stained for actin, tubulin, and DNA. The frequency and size of these bulges were greatly enhanced in anillin-depleted cells [9]. It is likely that both the pole- and the intercellular bridge-associated protrusions are dependent on changes in the contractility of the cell cortex and the extensive membrane remodeling that occurs during cytokinesis [25–27].

### Membrane behavior during S2 cell mitosis

Extensive literature supports the notion that the double membrane that forms the nuclear envelope in interphase cells is continuous with the ER. The outer nuclear membrane is connected to the ER through tubules or sheets and shares with the ER many proteins and lipids. In contrast, the inner nuclear membrane is biochemically distinct from the outer and contains hundreds of unique components, many of which are thought to interact with the nuclear lamina and the chromatin [28–31]. We found that interphase and prophase cells exhibit a DNM that surrounds the nucleus and a few patches of ER membranes that do not appear to be closely associated with the DNM. However, starting from early prometaphase, the membrane systems of S2 cells undergo a series of dramatic transformations, which are schematically summarized in Fig. 2a.

Our results clearly show that at the beginning of prometaphase, the nuclear envelope exhibits small regions made of QNM; the nuclear fenestration areas are always characterized by the presence of QNM. Following this initial appearance, the QNM forms along the entire nuclear envelope. Our data do not allow us to deduce a precise mechanism for QNM formation. Clearly, QNM assembly requires the formation of an additional double membrane along the DNM. In principle, this additional membrane could be synthesized de novo, either inside or outside the DNM. Alternatively, it could be a pre-existing ER membrane, which would first become closely apposed and then attach to the DNM. We favor this second possibility for several reasons. First, as mentioned earlier, there is continuity between the nuclear membrane and the ER, which are connected by tubules or sheets, a condition that would facilitate conjoining of the two double membranes. Second, we observed several early prometaphase cells showing an ER membrane running alongside the DNM and a few examples of close apposition of the ER membranes to the DNM (Additional file 2: Figure S2c and Additional file 5: Figure S5), two findings in line with the second alternative. Third, there are several examples of the apposition of ER membranes reported in the literature. The most pertinent one concerns *Drosophila* syncytial embryos, where during prometaphase, a second ER membrane forms around the DNM [20]. In another example, downregulation of the lipin homologue of *Caenorhabditis elegans* led to the formation of an extra ER layer in close association with the nuclear membrane and interfered with the disassembly of the nuclear envelope [32]. Stacked layers of ER membranes have also been observed in vertebrate cells overexpressing certain ER membrane proteins [33, 34] or infected with the African swine fever virus [35]. However, in all these systems, the ER sheets were not attached and did not show the type of adhesion observed in the QNM of S2 cells. Patches of QNM similar to those seen in S2 cells have been observed only in mammalian tumor cells undergoing mitosis but not in normal dividing cells [36, 37]. It has been also suggested that the QNM observed in these tumor cells is generated by the attachment of ER segments to the nuclear envelope [37].

We have shown that at the fenestration sites, the nuclear membrane exhibits multiple folds and invaginations that mostly comprise QNM. This dramatic deformation of the nuclear membrane is likely to be caused by the astral MTs that come into contact with the nucleus. Deep MT-dependent invaginations in the nuclear envelope have been also observed in vertebrate cells before NEBD, and it has been suggested that MT–nuclear envelope interactions trigger NEBD. However, NEBD can also occur in the absence of MTs, suggesting the existence of a MT-independent pathway for NEBD [30, 38–40]. These findings prompted us to investigate whether QNM formation requires an interaction with the astral MTs. To answer this question, we performed RNAi against the *cnn* gene that encodes a centrosome component essential for MT nucleation [41]. Consistent with previous studies [4], we found that RNAi-mediated Cnn depletion completely inhibits aster formation in S2 cells. A TEM analysis of Cnn-depleted cells revealed that several prometaphase cells exhibit a QNM (Additional file 9: Figure S9a), indicating that formation of this peculiar membrane structure does not depend on interactions between the DNM and the astral MTs. We also asked whether the QNM could form in the complete absence of MTs. We treated S2 cells for 3 h with colcemid and verified by tubulin immunostaining that this treatment completely depolymerizes both cytoplasmic and spindle MTs (not shown). A TEM analysis of colcemid-treated cells revealed that most of them are arrested in a prometaphase-like stage with some showing patches of QNM (Additional file 9: Figure S9b). Thus, QNM formation is independent of astral MTs and does not appear to require the presence of spindle or cytoplasmic MTs.

On progression through prometaphase, the inner double membrane of the QNM becomes swollen and progressively disassembles through vesiculation and the formation of cisternae (for an example, see Fig. 1c). Interestingly, nuclear envelope-derived cisternae have also been observed in mammalian cells undergoing NEBD [42–44]. The finding that both the inner component of the QNM and the mammalian nuclear envelope disassemble through vesicle formation supports the hypothesis that the inner membrane of the QNM corresponds to the DNM of interphase and PM1 cells. Disassembly of the inner membrane of the QNM is followed by the appearance of several openings in the nuclear envelope and the progressive transition from a closed to an open form of mitosis.

Measurements of the length of the different membrane types (ER membranes, DNM, QNM, and RDM) in 70 nm sections showed that total membrane length (counting the length of the QNM twice) in interphase, PM1, PM2, PM3, and PM4 cells is comparable. In contrast, the total membrane length in metaphase cells and anaphase cells is approximately 1.5-fold greater than in interphase or prometaphase cells (post hoc comparisons using the Tukey HSD test, *p* < 0.05) (Fig. 2b). This finding suggests that during prophase and prometaphase there is an extensive membrane remodeling with respect to interphase. During the short period that elapses between late prometaphase and metaphase, membrane remodeling is also extensive and is likely to be accompanied by additional membrane formation, a process that could be related with the need of membrane during cytokinesis.

An analysis of the distribution of the nuclear pore complexes (NPCs) in 70 nm sections showed that the interphase nuclei contain 21.8 ± 1.4 NPCs, with an average of one NPC per 0.98 μm of DNM. In the PM1 stage, the average number of NPCs in the DNM was drastically reduced (4.7 ± 0.9), with an average of one NPC per 4.4 μm of DNM. The QNM totally lacked NPCs crossing both double membranes. However, NPC-like structures were occasionally observed in the external double membrane of the QNM (Additional file 10: Figure S10a) and the RDM of PM3 and PM4 cells, and even in the ER membranes near the nuclear envelope (Additional file 10: Figure S10b). NPCs have been previously observed in the ER membranes that form the annulate lamellae in *Drosophila* syncytial embryos [20]. The ER membrane layers alongside the metaphase and anaphase spindles of S2 cells did not exhibit any NPC-like structures. NPCs reassembled concomitant with membrane formation around late anaphase B and telophase nuclei (Additional file 10: Figure S10c, d). Thus, as occurs in mammalian cells [30, 40], and consistent with previous work on *Drosophila* syncytial embryos [45], NPCs of S2 cells disassemble at the onset of mitosis and reassemble during telophase.

### Lamin behavior and the role of the QNM during S2 cell mitosis

**Fig. 6:**
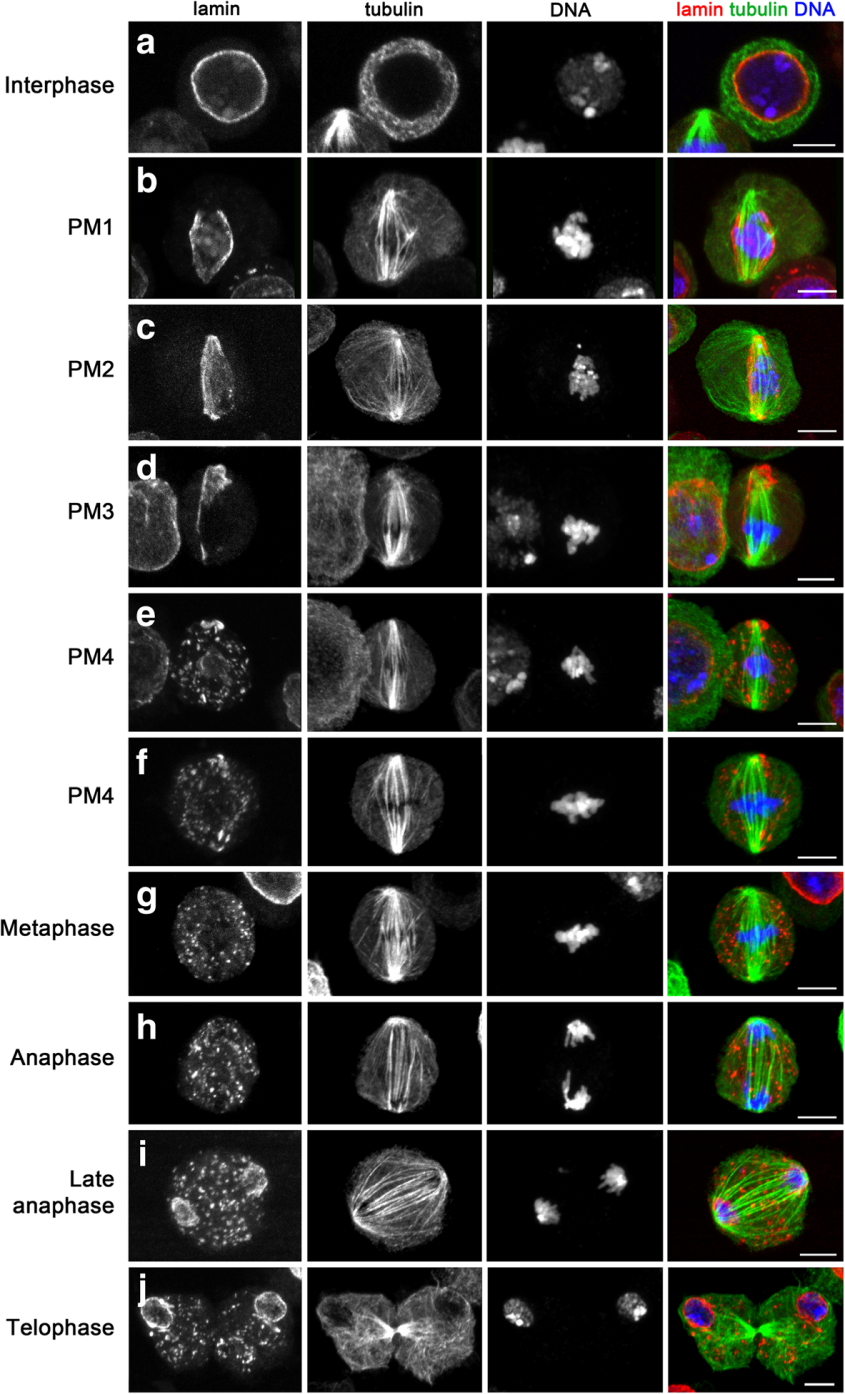
Lamin immunostaining recapitulates TEM observations. Cells were stained for lamin, tubulin, and DNA. A lamin meshwork encases the nucleus in interphase and early prometaphase cells (PM1). It begins to disintegrate in the PM2 stage and continues to disassemble during the PM3 stage. The nuclear lamina is completely disassembled in late prometaphase (PM4) and metaphase cells. Note that fragments of the nuclear lamina persist from prometaphase till late telophase. Scale bars: 5 μm

To integrate TEM observations, we stained dividing S2 cells that express tubulin-green fluorescent protein (GFP) with anti-lamin Dm0 [46–48] and anti-GFP antibodies, and examined them by confocal microscopy. The analysis of optical sections of these cells permitted visualization of the different stages of prometaphase identified by the TEM analysis. We analyzed 160 prometaphase cells. Of these, 16% displayed a continuous nuclear lamina except at fenestrations penetrated by MTs (Fig. 6b). These cells most likely correspond to the PM1 stage. 37% of these prometaphases showed an incomplete nuclear lamin meshwork (Fig. 6c, d) and are likely to correspond to the PM2 and PM3 stages. The remaining 47% of prometaphases examined displayed some remnants of the nuclear lamina and small patches of lamin dispersed throughout the cytoplasm (Fig. 6e, f). These cells are most likely in the PM4 stage. In metaphase cells, the chromosomes were not surrounded by any clear lamin-enriched structure. Thus, disintegration of the nuclear lamina occurs in the PM4 stage at the transition between prometaphase and metaphase.

Interestingly, after prometaphase, the lamin-enriched speckles, which are absent in interphase cells, persisted through metaphase, anaphase, and telophase (Fig. 6g–j). These lamin speckles have been observed previously in dividing S2 cells [49] but not in *Drosophila* embryonic or brain mitoses, or in male meiosis [50–53]. We do not know the composition or the function of these speckles, and they were not detected by the TEM analysis. They might be aggregations of lamin, other nuclear envelope proteins, and membrane that did not completely disassemble to facilitate reformation of the nuclear envelope in telophase. Indeed, starting from late anaphase and throughout telophase, we observed a lamin meshwork surrounding the two daughter nuclei (Fig. 6i, j). However, the lamin speckles persisted also after initial reformation of this nuclear envelope, raising the possibility that they could be used for nuclear envelope expansion in late telophase or during the ensuing early G1.

Collectively, our results suggest that *Drosophila* S2 cells evolved a QNM to maintain a closed mitosis up to mid/late prometaphase, to facilitate MT–kinetochore interactions during early prometaphase. Our observations suggest that the inner double membrane component of the QNM corresponds to the vertebrate nuclear membrane, which disintegrates at the beginning of prometaphase concomitantly with its associated lamin meshwork (NEBD). The formation of a QNM would maintain the continuity of the nuclear envelope, even when the inner double membrane component of the QNM disassembles, thus postponing NEBD from the beginning of prometaphase to mid/late prometaphase (stage PM4). The behavior of the lamin during prometaphase (Fig. 6) raises the question of whether the stability of the perinuclear lamin meshwork requires an interaction with the inner component of the QNM or whether it can also be stabilized by its outer component (the RDM). Our light microcopy observations (Fig. 6) appear to support the first alternative. However, further studies will be required to define the individual properties of the two double membranes of the QNM.

### Relationships between mitochondria and ER membranes

Studies carried out in both *Drosophila* and human cells have shown that membranous organelles (e.g., mitochondria) are excluded from the spindle region through a MT-independent mechanism [49, 54]. It has also been shown that soluble tubulin and proteins such as Megator and Mad-2 accumulate in the nucleoplasm soon after nuclear fenestration at the beginning of prometaphase and remain within the nucleus for approximately 10 min before spreading into the cytoplasm [49]. To explain this finding, it has been suggested that during early mitosis, these proteins are confined into the nucleoplasm by “spindle envelope” membranes that still surround the nucleus.

Our analyses showed the presence of a complex membranous system encasing the chromosomes during the early stages of prometaphase, and that this system becomes discontinuous after the PM3 stage. Nevertheless, our TEM photographs revealed that mitochondria are mostly excluded from the spindle/nuclear area, not only in the PM1, PM2, and PM3 prometaphase stages but also during the PM4 stage and the subsequent mitotic stages. We found that mitochondria are mostly associated with ER membranes that discontinuously surround the spindle during late prometaphase (PM4), metaphase, anaphase, and early telophase. These organelles were often found in close proximity to or attached to the ER membranes on both their inner and outer sides with respect to the spindle (Figs. 3 and 4, Additional file 2: Figure S2d, Additional file 6: Figure S6, Additional file 7: Figure S7, and Additional file 8: Figure S8b). Sometimes they were anchored to these membranes like bulbs on a garland (Additional file 8: Figure S8b). An intimate interconnection between mitochondria and ER has been observed in yeast and shown to be crucial for proper mitochondrial distribution during mitosis. However, it is unclear whether the ER and mitochondria also associate during animal cell mitosis [55], although recent work has shown that in dividing HeLa cells, mitochondria tend to localize within the ER folds [56].

Our results strongly suggest that the mitochondria and the ER physically interact during S2 cell mitosis and confirm the exclusion of these organelles from the spindle area [49]. However, our observations indicate that ER membranes do not form a continuous barrier against the invasion of the spindle region by mitochondria, as suggested previously [49]. Nonetheless, these membranes are likely to constitute a trap that captures these organelles, preventing their free diffusion within the dividing cells.

### Membrane behavior during mitosis in different *Drosophila* tissues

The most studied *Drosophila* mitotic system is the syncytial embryo, which is characterized by a series of extremely rapid and synchronous mitotic divisions. Membrane behavior in this system is similar but not identical to that just described for S2 cells. In embryonic divisions, a second membrane is also added just outside the nuclear membrane; this process begins in prometaphase near the fenestrated areas at the spindle poles and is completed at metaphase with the formation of two membrane layers that surround the entire spindle with the exception of the poles. These double membrane layers, often called the spindle envelope, persist during metaphase and anaphase. They contain ER markers, exhibit numerous irregular fenestrae but are never as closely apposed as the two double membranes that comprise the QNM of the S2 cells [20, 57, 58]. The single nuclear membrane regions around embryonic prometaphase nuclei exhibit disassembling NPCs but the two membrane layers of metaphase nuclei are virtually devoid of NPCs, which reassemble only in the membrane that surrounds telophase nuclei [20, 45]. Finally, in contrast with S2 cells, where the nuclear lamina disintegrates during prometaphase, in embryos, the lamina surrounds the spindles until late metaphase and dissolves just before anaphase [51, 52]. Thus, it appears that embryonic mitosis is more “closed” than S2 mitosis, consistent with the fact that embryonic nuclei are not surrounded by a plasma membrane.

The spindles of *Drosophila* larval brain neuroblasts are surrounded by a single fenestrated membrane. As in embryos, this membranous structure persists until late anaphase (TEM observations by A. T. C. Carpenter, personal communication). In addition, it has been shown that from metaphase to late anaphase of brain neuroblasts, the phosphatidylinositol transfer protein Giotto is enriched at an intracellular structure, which in location and shape corresponds to the membranous spindle envelope observed by TEM [59]. Phosphatidylinositol transfer proteins are enriched at cell membranes, where they bind lipid monomers, facilitating their transfer between different membrane compartments [60]. Consistent with these results, in vivo studies have shown that GFP-lamin marks the nuclear envelope from prophase till anaphase, although the anaphase GFP signal is weaker than those observed in prometaphase and metaphase cells [52]. These observations suggest that neuroblast mitosis has a similar membrane behavior as mitosis in syncytial embryos. Thus, S2 cell mitosis appears to be more “open” than both neuroblast and syncytial embryo mitosis.

The few prior studies performed in S2 cells have not provided details on the behavior of the intracellular membrane during mitosis. They only reported that a membrane-based nuclear envelope disappears just prior to metaphase plate formation [61]. A study on the heat shock effects in Kc cells, another *Drosophila* cell line of embryonic origin, showed that the spindles of cells not treated with heat are surrounded by two membrane layers that run adjacent to each other but do not exhibit areas of close apposition [21]. Thus, they are somewhat similar to the paired double membranes that encase embryonic divisions. Spindle envelopes containing a variable number of membrane layers have been described in *Drosophila* spermatocytes [62, 63] and in mitotic and meiotic spindles of several species of Diptera and Lepidoptera [64, 65]. However, the complex intracellular membrane behavior we observed in S2 cell mitosis has not been described in any of these systems. Moreover, published images do not show closely apposed membrane layers comparable to the QNM we observe in S2 cells [21, 62, 64, 65].

### MT behavior and MT–kinetochore interactions in S2 cells

**Fig. 7:**
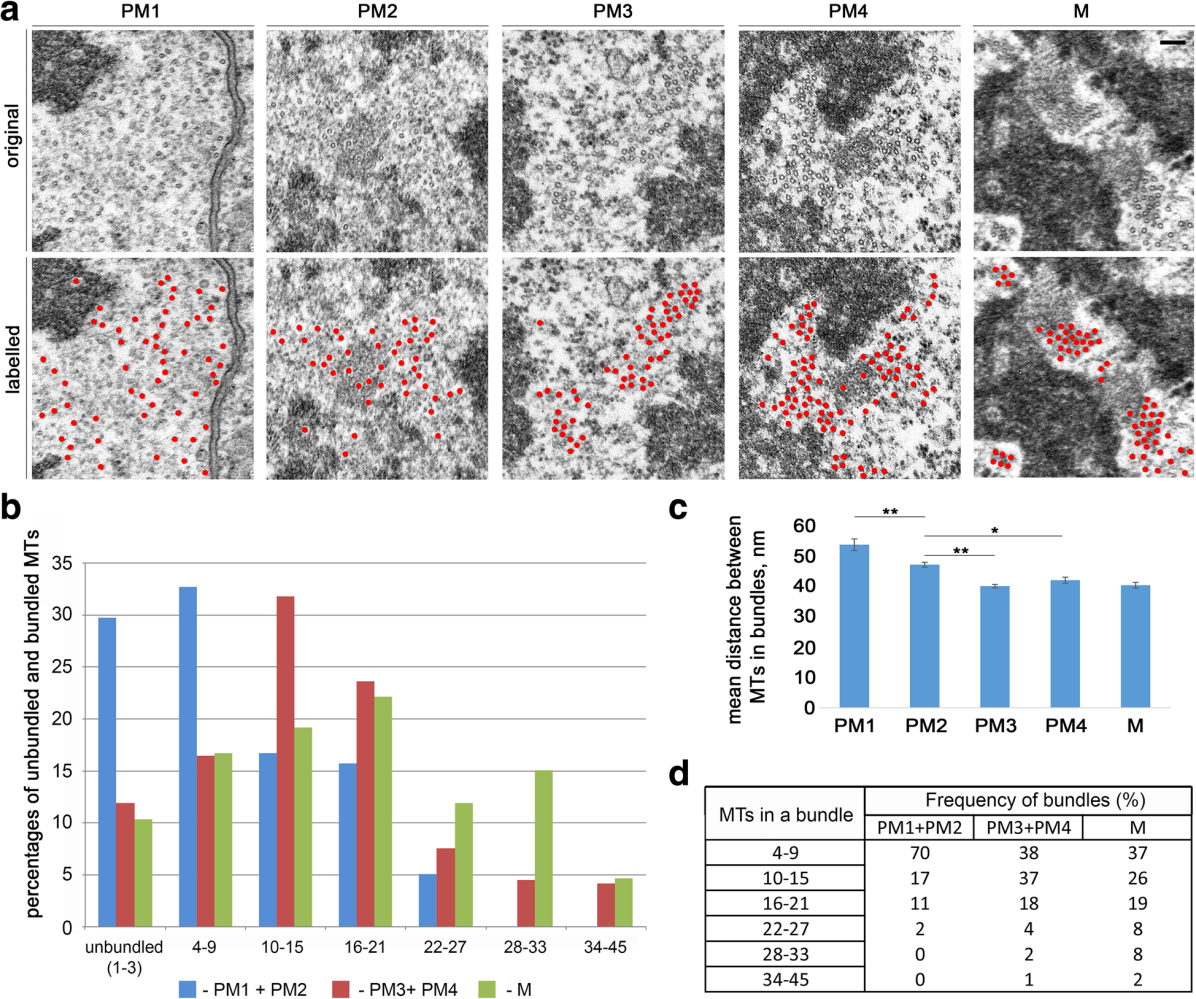
MT organization in prometaphase and metaphase S2 cells. **a** Examples of transverse sections of MTs in prometaphase and metaphase cells. In the bottom panels, MTs are pseudo-colored in red. In the PM1 cell shown, MTs are not bundled. In the PM2, PM3, PM4, and metaphase (M) cells, MTs are organized in bundles, and the average distance between bundled MTs decreases during the progression from early prometaphase to metaphase. Scale bar: 0.1 μm. **b** Frequencies of unbundled MTs (1–3 MTs) and of MTs included in bundles of different sizes (4–9, 10–15, 16–21, 22–27, 28–33, and 34–45 MTs) observed in transverse sections through the chromosomes of prometaphase and metaphase cells. All MT bundles were taken into account, including those outside the metaphase plate. **c** Quantification of the distances between MTs within bundles (10 bundles were analyzed for each stage). The average distances observed in PM3, PM4, and metaphase (M) cells are significantly smaller than those seen in PM1 and PM2 cells (* and **, *p* < 0.05 and 0.01, respectively; ANOVA with post-hoc Tukey HSD). **d** Frequencies of MT bundle sizes (number of MTs per bundle) from early prometaphase to metaphase (74, 102, and 101 bundles were analyzed for the PM1 + PM2, PM3 + PM4, and M stages, respectively). M metaphase

**Fig. 8:**
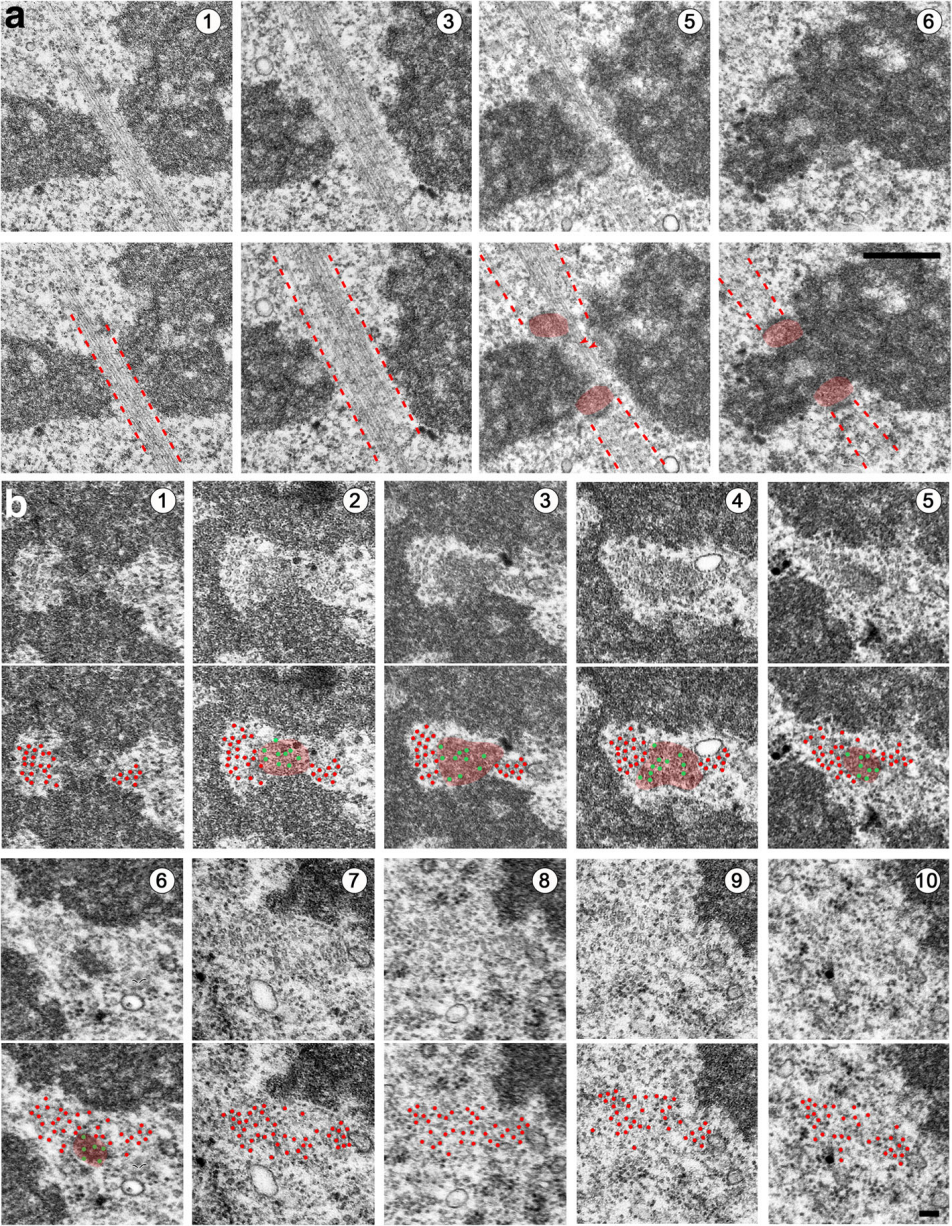
Organization of MT bundles in late prometaphase/metaphase S2 cells. **a** Longitudinal serial sections showing a large MT bundle comprising MTs that are attached to kinetochores (k-fibers) and MTs that run laterally to kinetochores. In the bottom panels, the dotted red lines delimit the MT bundles and the kinetochores are pseudo-colored in red. **b** Transverse serial sections through a large MT bundle comprising kinetochore-attached MTs (k-fiber) and two lateral MT bundles. The first and third series of panels show the original images. In the second and fourth series of panels, kinetochores and most MTs are pseudo-colored in red. MTs embedded into the kinetochore are less clear than the other MTs in the section and are tentatively pseudo-colored in green. Scale bars: **a**, 1 μm; **b**, 0.1 μm

To analyze MT behavior and MT–kinetochore relationships, we examined both single and serial sections of mitotic cells cut either longitudinally or transversely with respect to the spindle axis. We first consider transverse sections through prometaphase and metaphase chromosomes. The PM1, PM2, PM3, PM4, and metaphase stages were recognized by the degree of chromatin compaction and by the intracellular membrane pattern. In the early stages of prometaphase, MTs are rather sparse but tend to aggregate into bundles as prometaphase proceeds (see Additional file 3: Figure S3 for overall examples of MT distribution in transverse sections; detailed examples are shown in Figs. 7a and 8b). To characterize the MT bundles in transverse sections at different mitotic stages, we measured the average distance between the MT centers within each bundle (see “Methods” for the measuring procedures). We considered to be MT bundles geographically isolated MT aggregates, in which the average distance between the MTs is between 35 and 50 nm. Aggregates of two or three MTs were not considered to be bundles. This analysis showed that the percentage of bundled MTs (total number of MTs included in bundles/total number of bundled plus unbundled MTs) increases during the progression from early prometaphase to metaphase (Fig. 7b). Conversely, the distance between MTs within the bundles decreases when cells progress from early prometaphase to metaphase (Fig. 7c). The average inter-MT distance observed in PM3 bundles is significantly lower than that seen in PM2 bundles, which in turn is significantly lower than that observed in PM1 bundles (post hoc comparisons using the Tukey HSD test, *p* < 0.01). The inter-MT distances within the PM3, PM4, and metaphase bundles are not significantly different. In addition, during the progression from early prometaphase to metaphase, there is an increase in the frequencies of large bundles containing 10–15, 16–21, and >21 MTs (Fig. 7d). The largest MT bundles (34–45 MTs) were found only in PM3, PM4, and metaphase cells (Fig. 7d).

We were not able to visualize mature kinetochore structures before the PM4 stage. In the PM1–PM3 stages, the kinetochores are oblong and never show end-on-attached MTs. In PM4 cells, kinetochores have an arched shape similar to that of their metaphase counterparts and often display end-on attached MTs (Additional file 4: Figure S4). These observations are consistent with the finding that at mitotic entry, human kinetochores appear as large crescents and compact into the discrete kinetochore structures only after the attachment to MTs in an end-on fashion [66]. In agreement with previous findings [61], longitudinal sections of metaphase and early anaphase S2 cells showed that kinetochores with attached MTs are arched structures that show only one electron-dense layer (Figs. 3b, 4a, Additional file 4: Figure S4, and Additional file 6: Figure S6a) and fail to exhibit the two typical layers that characterize vertebrate kinetochores. However, after MT depolymerization with colchicine, S2 cell kinetochores exhibit an inner layer and an outer layer, suggesting that the presence of MTs distorts the kinetochore structure [61].

An analysis of longitudinal and transverse serial sections (Fig. 8) showed that k-fibers contain on average 12 ± 1 MTs, consistent with previous ultrastructural studies on S2 cells [61]. We also observed MT bundles running laterally to the kinetochores (lateral bundles). To define the number of MTs in lateral bundles, we analyzed in detail eight transverse sections through metaphase chromosomes (Fig. 8 and Additional file 11: Figure S11). When serial sections were available, we examined the section that was cutting the highest number of kinetochores or inter-kinetochore (centromeric) chromatin regions. Notably, in all these sections, we could distinguish two separate MT bundles, which often contained different numbers of MTs. In sections through the kinetochore or the centromeric chromatin, most lateral bundles contained either 10–15 or 22–33 MTs. In regions not including the kinetochores or the chromatin, we observed bundles containing 28–45 MTs (Fig. 8b and Additional file 11: Figure S11). In all cases where we could examine serial sections, these large MT bundles clearly comprised both kinetochore fibers and lateral bundles (see Fig. 8b for an example).

**Fig. 9:**
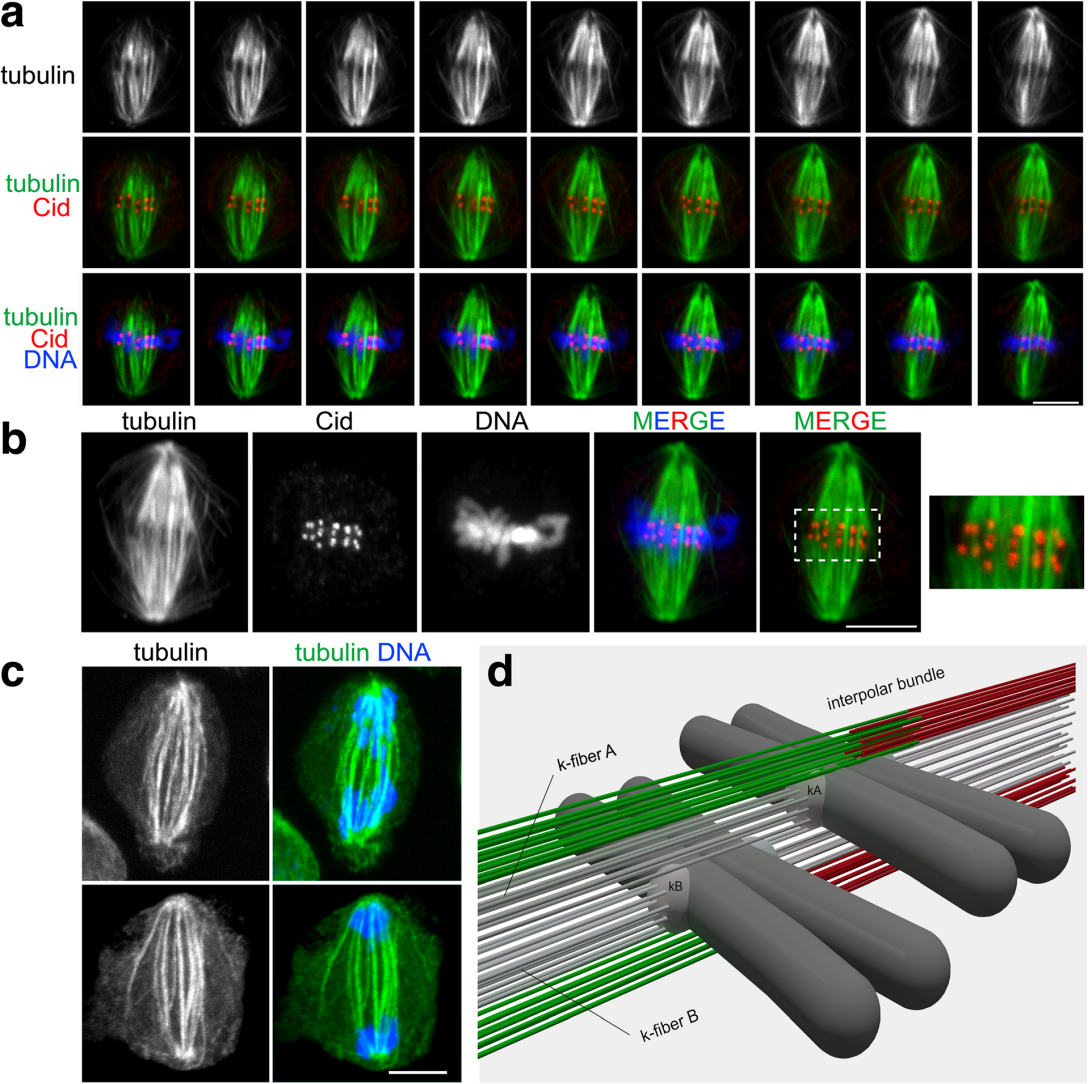
Organization of MT bundles in metaphase S2 cells. **a, b** Serial optical sections (**a**) and maximum projection (**b**) of a metaphase cell stained for tubulin, the centromere marker Cid, and DNA (DAPI). Some MT bundles are running continuously between the two spindle poles without encountering the centromeres (interpolar bundles), while other bundles end at kinetochores (k-fibers). Note that MT bundles attached to different kinetochores and interpolar bundles are closely apposed (and/or intermingled) in the regions between the centromeres and the spindle poles. Scale bars: 5 μm. **c** Maximum intensity projections of confocal images of early (top) and late (bottom) anaphase cells showing interpolar MT bundles. Scale bar: 5 μm. **d** A model for the MT–kinetochore interaction during late prometaphase and metaphase. The model depicts a portion of a late prometaphase/metaphase cell that includes only two chromosomes. Grey MT bundles are k-fibers. Green and red MTs are interpolar MT bundles running laterally to kinetochores. The scheme does not imply that the k-fibers are as numerous as the interpolar MT bundles. k-fibers A and B are attached to the kinetochores of two neighboring chromosomes. Note that k-fiber A is a lateral MT bundle for kinetochore B (kB)

These observations raise a question about the nature of the lateral MT bundles. Do they comprise antiparallel MTs running from pole to pole with a region of overlap at the cell equator, or are they instead MTs that terminate in another kinetochore located proximally or distally with respect to the sectioning plane? Our TEM analyses clearly showed the existence of long pole-to-pole MT bundles (Additional file 12: Figure S12) but did not provide sufficient information on the behavior of all lateral MT bundles. To address this question, we made mitotic preparations stained for tubulin, Cid, and DNA and examined them by confocal microscopy. Cid, the *Drosophila* homologue of CENP-A, is a specific component of centromeric heterochromatin that is routinely used as a kinetochore marker [67]. Analysis of optical sections and three-dimensional computer-reconstructed images of late prometaphase cells and metaphase cells showed that they contain pole-to-kinetochore MT bundles (k-bundles), bundles emanating from one of the poles and terminating beyond the metaphase plate without reaching the opposite pole (these bundles are mostly located at the spindle periphery), and interpolar bundles that appear to run from pole to pole and have approximately the same size as the k-bundles. We found that some of the bundles, running adjacent to a kinetochore, were attached in an end-on fashion to a different kinetochore, while others appear to run towards a spindle pole without encountering another kinetochore (Fig. 9a, b). Examination of optical sections of 89 metaphase cells led us to estimate that they contain from four to 10 interpolar MT bundles with an average of 6.4 bundles per cell. This finding was corroborated by the analysis of 16 anaphase figures, which showed from five to 11 interpolar bundles, with an average of 7.2 bundles per cell (Fig. 9c). The precision of this analysis is limited since we were not able to visualize the overlapping zone of the antiparallel MTs in the interpolar bundles. In human cells, this zone is marked by the PRC1 protein [68], whereas Feo, the *Drosophila* homologue of PRC1, does not mark the interpolar MT bundles in metaphase cells, but concentrates in the MT overlap zone starting from late anaphase [69]. An analysis of PRC1 localization in metaphase HeLa cells revealed that nearly all PRC1-enriched zones of interpolar MT bundles are associated with a kinetochore pair, suggesting a one-to-one ratio between kinetochore fibers and interpolar MT bundles [68].

Our confocal and TEM observations are summarized in a three-dimensional model of MT organization during late prometaphase and metaphase (Fig. 9d). This model provides an explanation for the different numbers of MTs in the bundles. We propose that bundles of 10–15 MTs are either kinetochore or pole-to-pole bundles, while bundles of 22–33 MTs are likely to comprise different combinations of these two bundle types. The largest MT bundles observed in metaphase cells (comprising up to 45 MTs) probably comprise both kinetochore fibers and interpolar MT bundles, and might reflect the presence of imperfectly aligned metaphase chromosomes with tandemly arranged kinetochores along the spindle axis. This type of kinetochore arrangement in tightly packed metaphase plates is common in *Drosophila* S2 cells (see Fig. 9a, b) and has been observed previously [70].

The associations between k-fibers of 10–15 MTs and large lateral MT bundles (comprising 7–33 MTs) seem to be peculiar to *Drosophila* S2 cells. MT bundles running laterally to kinetochores have been previously observed by TEM in both longitudinal and cross sections of several spindle types, including those of rat kangaroo PTK cells [71, 72] and HeLa cells [73–75], as well as in spindles assembled from *Xenopus* egg extracts [76]. However, in all cases, in contrast to S2 cells, the lateral MT bundles appeared to contain many fewer MTs than the kinetochore fibers.

There is evidence that the lateral MT bundles play important roles in chromosome segregation. The most extreme case has been described in the acentrosomal meiotic spindles of *C. elegans* females, where kinetochores are not required for chromosome segregation and chromosomes segregate due to motor protein-mediated interactions with lateral MT bundles [77–79]. In *Drosophila* female meiosis, lateral kinetochore–MT attachments are sufficient for prometaphase chromosome movements but end-on kinetochore–MT attachments are required for homolog bi-orientation, indicating that both lateral and end-on kinetochore–MT interactions cooperate to ensure accurate chromosome segregation [80]. Also, in centrosome-containing spindles of mammalian cells, lateral kinetochore–MT interactions have relevant roles in mitotic division. For example, mono-oriented chromosomes can congress to the metaphase plate by moving alongside kinetochore fibers attached to other chromosomes, exploiting the motor activity of the CENP-E kinesin-like protein [81]. In addition, k-fibers of mammalian cells that have lost a direct connection to the pole can nonetheless promote chromosome segregation through dynein-mediated lateral interactions with the k-fibers of adjacent chromosomes or other non-kinetochore MTs [82]. Consistent with these results, it has been shown that interpolar MT bundles can physically associate with k-fibers, forming a bridge between two sister kinetochore fibers. These bridging MT bundles (called bridging fibers) are thought to balance the forces acting at kinetochores and to contribute to both spindle shaping and chromosome movement [83–85]. Based on these studies, we suggest that the peculiar organization of MTs observed in the S2 metaphase spindle (Fig. 9) reflects lateral interactions between kinetochores and both k-fibers and interpolar MTs. These interactions are likely to be functionally important in ensuring accurate chromosome segregation.

## Conclusions

We have shown that the spindles of S2 cells comprise multiple discrete MT bundles that are likely to interact with each other to ensure proper spindle function. This spindle organization appears to be slightly different from that observed in vertebrate cells. However, we do not know whether it is different from the structure of *Drosophila* embryonic or neuroblast spindles, which are currently incompletely characterized at the ultrastructural level. We have also shown that dividing S2 cells exhibit a peculiar intracellular membrane behavior, which has not previously been seen during mitosis of vertebrate cells, *Drosophila* embryonic nuclei, or *Drosophila* larval neuroblasts. Overall, our observations clearly show that besides its characteristic membrane behavior, mitosis of S2 cells is more “open” than in its embryonic or neuroblast counterparts. This finding raises the interesting question of whether this feature of S2 mitosis is also present in the late (cellularized) embryonic cells from which the S2 lineage derives, or was instead acquired during the process of S2 cell immortalization. Distinguishing between these alternatives and defining whether different *Drosophila* cell types exhibit variations in their mitotic structures will require further ultrastructural studies in additional fly tissues and their derivative cell lines [86].

## Methods

### Cell culture and RNA interference

The origin of the *Drosophila* S2 cell line used here has been described previously [87]. S2 cells and S2 cells expressing tubulin-GFP were maintained at 25 °C in Shields and Sang M3 medium (Sigma) supplemented with 20% heat-inactivated fetal bovine serum (Invitrogen); all cells were free from mycoplasma contamination. The cell density was kept below 6–8 × 10^6^ cells/ml to avoid the formation of cell aggregates. *cnn* dsRNA production and RNAi treatments were carried out according to [4]. dsRNA-treated S2 cells were grown for 5 days at 25 °C, and then processed for TEM analysis.

### Transmission electron microscopy

To prepare the S2 cells for the ultrastructural analysis, we used the protocol described in detail by [87]. Briefly, a cell pellet was fixed in 2.5% glutaraldehyde in 0.1 M sodium cacodylate buffer for 1 h at room temperature, post-fixed for 1 h in a 1% aqueous solution of osmium tetroxide containing a few crystals of potassium ferricyanide (K_3_[Fe(CN)_6_]), and then incubated overnight at 4 °C in a 1% aqueous solution of uranyl acetate. The next day, the specimens were dehydrated in ethanol series and then in acetone, and embedded in Agar 100 Resin (Agar Scientific, Essex, UK). Complete polymerization of samples was achieved by keeping them in an oven for 3 days at 60 °C. Ultrathin (70 nm) sections were obtained with a Leica ultracut ultra-microtome (Leica ultracut UCT, Vienna, Austria). Sections were examined with a JEOL JEM-100SX transmission electron microscope at 60 kV in the Inter-institutional Shared Center for Microscopic Analysis of Biological Objects (Institute of Cytology and Genetics, Novosibirsk, Russia). The images obtained were slightly modified in Photoshop CS5 using levels, brightness, and contrast tools. Three-dimensional reconstructions of prometaphase cells were obtained using the free editor Reconstruct [88].

### Measurements with ImageJ

The length of membranes (DNM, RDM, QNM, and ER) in S2 cells at different mitotic stages was measured with ImageJ software [89], using the simple freehand selection tool and measure function. We considered only longitudinal or slightly oblique longitudinal sections. To measure the total length of membranes in each section, we drew a freehand line between each lipid bilayer starting from one end to the other, and then summed the measures obtained for individual membranes. The data obtained for each mitotic stage were compared using one-way ANOVA with a post-hoc Tukey HSD test. The width of membranes and electron-dense material in the QNM was also measured using the line tool by drawing a line perpendicular to the parallel membranes. Ten different cells were used for the QNM measurements.

The distance between MTs within a bundle at different mitotic stages was also measured using the ImageJ software. For each mitotic stage, we analyzed 10 transversely cut bundles containing at least 10 MTs. For each bundle, we measured the radial distances between the MT at the center of the bundle and its neighbor MTs. Measurements were taken from MT center to MT center and the mean distance was quantified (a similar method was used by [90]). Data obtained for different mitotic stages were compared using one-way ANOVA with a post-hoc Tukey HSD test.

### Confocal microscopy observations

In total, 2 × 10^6^ S2 cells were centrifuged at 200×*g* for 5 min, washed in 2 ml of PBS (Sigma), and fixed for 10 min in 2 ml of 3.7% formaldehyde in PBS. Fixed cells were spun down by centrifugation at 200×*g* for 5 min, resuspended in 500 μl of PBS, and placed onto a clean slide using a Cytospin™ 4 centrifuge (Thermo Fisher Scientific) at 900 rpm for 4 min. The slides were then immersed in liquid nitrogen, washed in PBS, incubated in PBT (PBS + 0.1% TritonX-100) for 30 min and then in PBS containing 3% bovine serum albumin for 30 min. The following primary antibodies were used for immunostaining (all diluted in PBT): mouse monoclonal anti-α-tubulin (1:600, Sigma, T6199), mouse monoclonal anti-Lamin Dm0 (1:300, Developmental Studies Hybridoma Bank, ADL67.10), rabbit polyclonal anti-Cid (1:300, Abcam, ab10887), and rabbit polyclonal anti-GFP (1:200, Invitrogen, A11122). These primary antibodies were detected by incubation for 1 h with FITC-conjugated anti-mouse IgG (1:30, Sigma, F8264), Alexa Fluor 568-conjugated anti-mouse IgG (1:500, Invitrogen, A11031), Alexa Fluor 488-conjugated anti-rabbit IgG (1:300, Invitrogen, A11034), and Alexa Fluor 568-conjugated anti-rabbit IgG (1:350, Invitrogen, A11077). All slides were mounted in Vectashield medium with DAPI (Vector Laboratories, H-120) to stain DNA and reduce fading of fluorescence. Confocal immunofluorescent images were obtained on a Zeiss LSM 710 confocal microscope, using an oil immersion 100×/1.40 plan-apo objective and the ZEN 2012 software.

## Additional files

Additional file 1: **Figure S1.** Examples of interphase and early prophase cells. **a**, **b** Interphase cells showing round nuclei encased by a continuous double nuclear membrane (DNM). **c** A possible early prophase cell characterized by an undulated nuclear membrane. The asterisk indicates the cell region shown at higher magnification. Scale bars: **a** (left image), **b** and **c**, 5 μm; **a** (right image), 1 μm. (TIF 17489 kb)

Additional file 2: **Figure S2.** Serial sections of prometaphase and metaphase cells and 3D reconstruction of intracellular membrane organization. **a** Serial sections (numbers specify the section shown) of a PM1 cell showing the QNM in the area of nuclear fenestration and a normal DNM along most of the nuclear envelope. Note the association of the chromatin with the DNM (red arrows). **b** Serial sections of a PM2 cell showing a nuclear envelope mostly composed of QNM, and ER membranes laying outside the nuclear envelope. **c** Serial sections of a PM3 cell showing partial disassembly of the inner membrane component of the QNM through a vesiculation process. **d** Sections of a metaphase cell showing complete disassembly of the QNM, and ER membrane stacks along the spindle. Note the mitochondria associated with the ER membranes. Scale bars: 1 μm. (TIF 14176 kb)

Additional file 3: **Figure S3.** Overall MT distribution in transverse sections of S2 cells at different prometaphase stages. In the right images, the MTs and MT bundles of PM1 **a**, PM2 **b**, PM3 **c**, and PM4 **d** cells are encircled with a red line. Note that on progression through prometaphase, both the size (number of MTs) and the density (distance between MTs) of MT bundles increase. Scale bars: 1 μm. (TIF 22315 kb)

Additional file 4: **Figure S4.** Kinetochore structure in different mitotic phases of S2 cells. In PM1 and PM2 cells, kinetochores (pseudo-colored in red) have an oblong appearance and do not appear to interact with MTs in an end-on fashion. Only a fraction of PM3 kinetochores show a limited end-on MT binding. Kinetochores of PM4, metaphase (M), and early anaphase (A) cells exhibit an arched structure and show end-on attached MTs. Scale bar: 0.1 μm. (TIF 10897 kb)

Additional file 5: **Figure S5.** The ER membranes occasionally become closely apposed to the nuclear envelope. The cell shown is in the PM3 stage, as its nuclear envelope comprises both regions of QNM and regions of RDM. The cell also contains ER membranes, which in some cases (arrowheads) become closely apposed to the nuclear envelope forming a structure that is apparently identical to a QNM. Scale bar: 1 μm. (TIF 12005 kb)

Additional file 6: **Figure S6.** Additional examples of metaphase S2 cells. **a** Longitudinal section showing stacks of parallel ER membranes and kinetochores with k-fibers (arrowheads in the magnified image). **b** Cross section through a metaphase plate shown at different magnifications. Asterisks in the right image indicate the regions magnified in the insets. The **b'** inset shows a kinetochore and associated MTs (both pseudo-colored in red). The **b"** inset shows two MT bundles that might be either k-fibers or interpolar MT bundles (see Fig. 9 and Additional file 12: Figure S12). Scale bars: left images, 5 μm; right images, 1 μm; insets, 0.1 μm. (TIF 18615 kb)

Additional file 7: **Figure S7.** An additional example of an S2 cell in late anaphase. Shown is the initial assembly of the central spindle. Note the apparently antiparallel MTs overlapping at the center of the cell (arrowhead in the magnified image on the right). Scale bars: left image, 5 μm; right image, 1 μm. (TIF 7426 kb)

Additional file 8: **Figure S8.** Additional examples of telophase S2 cells. **a** Early telophase showing chromosomes partially surrounded by a double membrane, protrusions from the cell wall, and MT bundles at the center of the cell. **b** Early telophase with large protrusions from the cell wall and mitochondria attached to the ER membranes. **a**, **b** Asterisks indicate the cell regions shown at higher magnifications on the right. **c**, **d** Magnified images of late telophase intercellular bridges showing multiple membrane blebs and overlapping MTs in the middle of the bridges. Scale bars: 1 μm; except left images in A and B, 5 μm. (TIF 22942 kb)

Additional file 9: **Figure S9.** The QNM forms in the absence of astral microtubules. **a** A prometaphase cell in which the nucleation of astral MTs is completely suppressed by RNAi-mediated depletion of the centrosome component Cnn. It exhibits a QNM comparable to that observed in cells in which aster formation is not inhibited. **b** A prometaphase-like cell from a culture treated for 3 h with colcemid shows patches of QNM. The asterisks indicate the cell regions shown at higher magnification on the right. Scale bars: 1 μm. (TIF 15859 kb)

Additional file 10: **Figure S10.** Examples of ectopic nuclear pores in the outer component of the QNM and in the ER membranes. **a** PM3 cell showing an NPC in the outer component of the QNM (arrowheads in the magnified image shown on the right). **b** PM2 cell showing NPCs in the RDM and ER membrane approaching the nuclear envelope. **a, b** Asterisks indicate the cell regions shown at higher magnifications on the right. **c, d** Enlarged images showing NPC formation in the membranes surrounding **c** late anaphase and **d** telophase chromosomes. Scale bars: **a, b** left images, 1 μm; **a, b** right images, **c, d**, 0.1 μm. (TIF 19102 kb)

Additional file 11: **Figure S11.** Analysis of the lateral MT bundles in metaphase S2 cells. **a** MT bundles observed in a transverse section through the metaphase chromosomes of S2 cells. Only MT bundles within the metaphase plate were considered. Bundles outside this area, often containing four to nine MTs, were not taken into account. In sections through the kinetochore or the centromeric chromatin, we were always able to distinguish two separate MT bundles (indicated as bundle 1 and 2), which often contain different numbers of MTs. **b** Distribution of the MT bundles of different sizes (number of MTs) obtained by plotting the data shown in **a**. (TIF 216 kb)

Additional file 12: **Figure S12.** Long MT bundles in a metaphase S2 cell. Shown is a long MT bundle running between the two spindle poles without interruptions (asterisks). Scale bar: 1 μm (TIF 12551 kb)

## Abbreviations

DNM: Double nuclear membrane
dsRNA: Double-stranded RNA
ER: Endoplasmic reticulum
GFP: Green fluorescent protein
mRNA: Messenger RNA
MT: Microtubule
NEBD: Nuclear envelope breakdown
NPC: Nuclear pore complex
PBS: Phosphate-buffered saline
QNM: Quadruple nuclear membrane
RDM: Residual double membrane
RNAi: RNA interference
TEM: Transmission electron microscopy
