# Supplementary figures and images for: Ultrastructural analysis of mitotic Drosophila S2 cells identifies distinctive microtubule and intracellular membrane behaviors
Source: BMC Biol. 2018 Jun 15;16:68. doi: 10.1186/s12915-018-0528-1 (PMC6003134; doi:10.1186/s12915-018-0528-1)

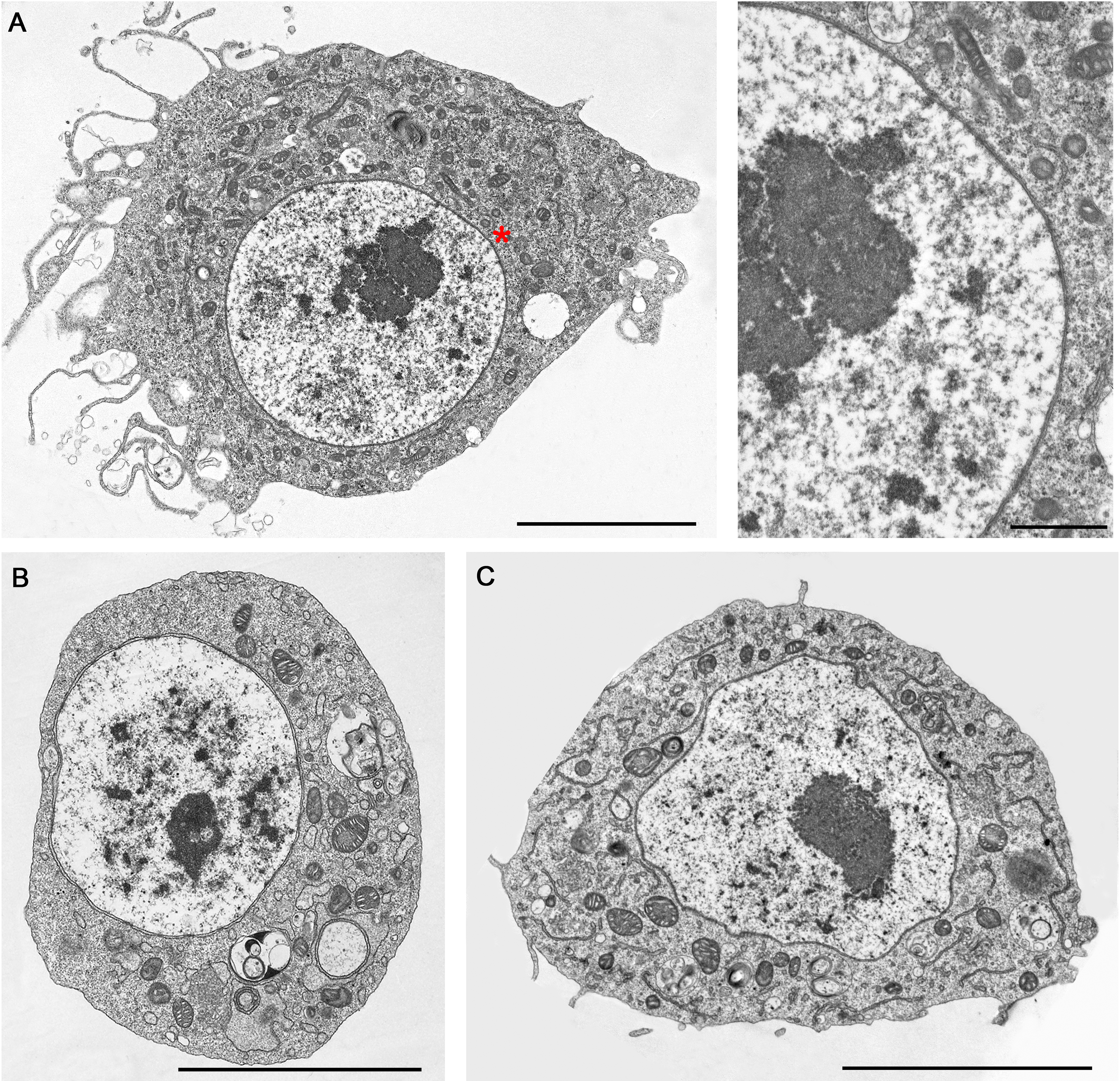

Supplement: Supplementary file 1 — Figure S1. Examples of interphase and early prophase cells. a, b Interphase cells showing round nuclei encased by a continuous double nuclear membrane (DNM). c A possible early prophase cell characterized by an undulated nuclear membrane. The asterisk indicates the cell region shown at higher magnification. Scale bars: a (left image), b and c, 5 μm; a (right image), 1 μm. (TIF 17489 kb) [file 12915_2018_528_MOESM1_ESM.tif]

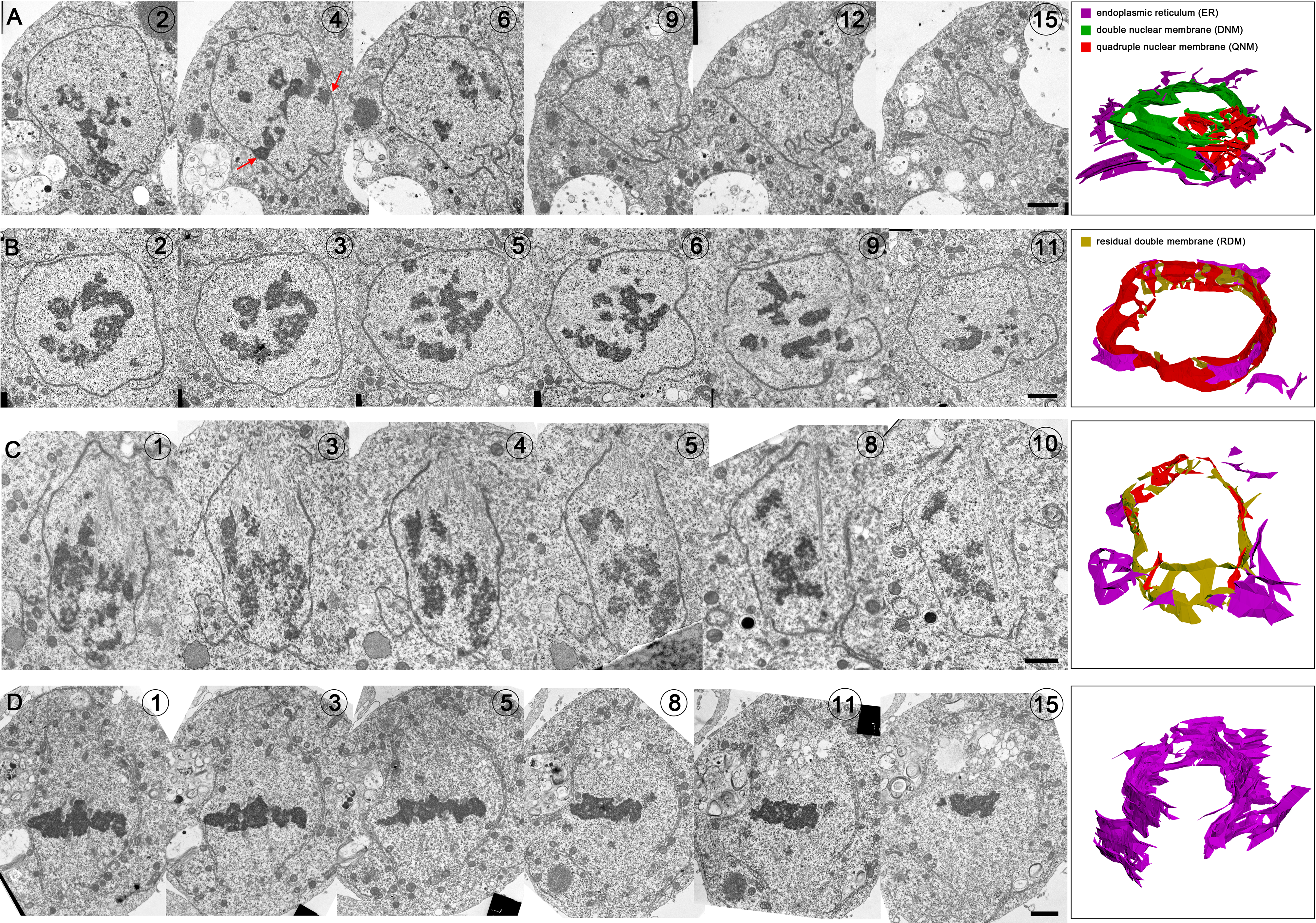

Supplement: Supplementary file 2 — Figure S2. Serial sections of prometaphase and metaphase cells and 3D reconstruction of intracellular membrane organization. a Serial sections (numbers specify the section shown) of a PM1 cell showing the QNM in the area of nuclear fenestration and a normal DNM along most of the nuclear envelope. Note the association of the chromatin with the DNM (red arrows). b Serial sections of a PM2 cell showing a nuclear envelope mostly composed of QNM, and ER membranes laying outside the nuclear envelope. c Serial sections of a PM3 cell showing partial disassembly of the inner membrane component of the QNM through a vesiculation process. d Sections of a metaphase cell showing complete disassembly of the QNM, and ER membrane stacks along the spindle. Note the mitochondria associated with the ER membranes. Scale bars: 1 μm. (TIF 14176 kb) [file 12915_2018_528_MOESM2_ESM.tif]

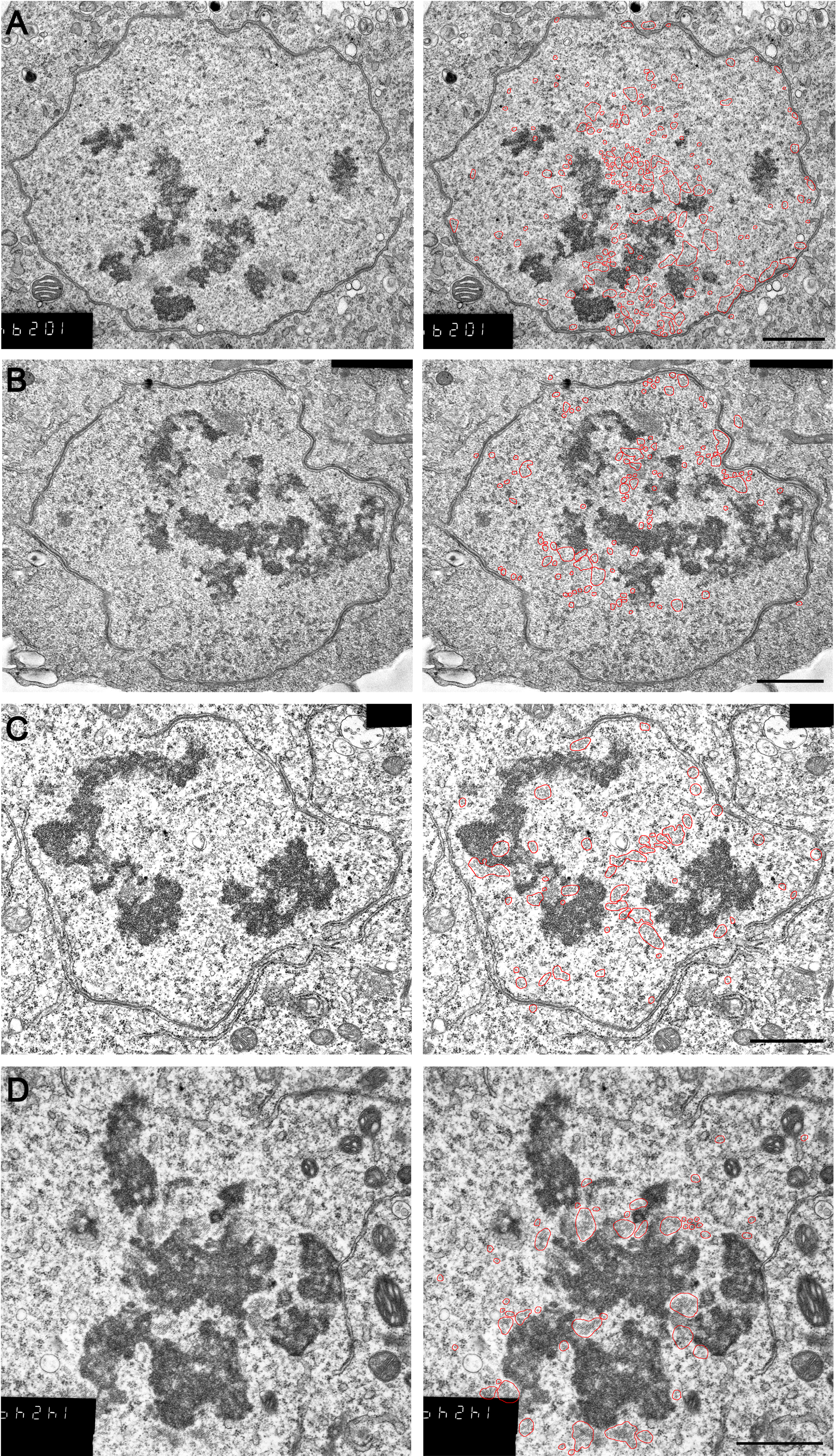

Supplement: Supplementary file 3 — Figure S3. Overall MT distribution in transverse sections of S2 cells at different prometaphase stages. In the right images, the MTs and MT bundles of PM1 a, PM2 b, PM3 c, and PM4 d cells are encircled with a red line. Note that on progression through prometaphase, both the size (number of MTs) and the density (distance between MTs) of MT bundles increase. Scale bars: 1 μm. (TIF 22315 kb) [file 12915_2018_528_MOESM3_ESM.tif]

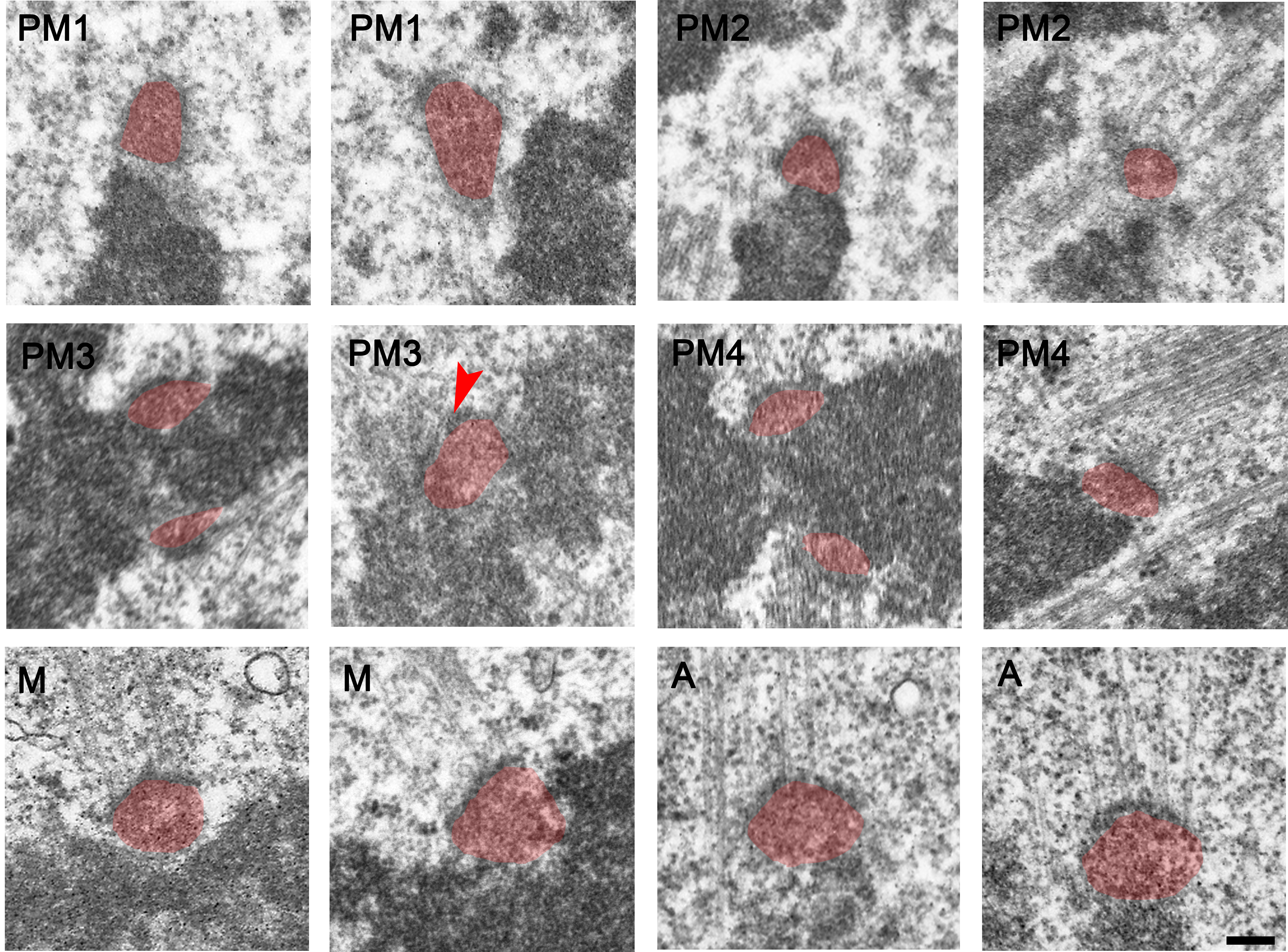

Supplement: Supplementary file 4 — Figure S4. Kinetochore structure in different mitotic phases of S2 cells. In PM1 and PM2 cells, kinetochores (pseudo-colored in red) have an oblong appearance and do not appear to interact with MTs in an end-on fashion. Only a fraction of PM3 kinetochores show a limited end-on MT binding. Kinetochores of PM4, metaphase (M), and early anaphase (A) cells exhibit an arched structure and show end-on attached MTs. Scale bar: 0.1 μm. (TIF 10897 kb) [file 12915_2018_528_MOESM4_ESM.tif]

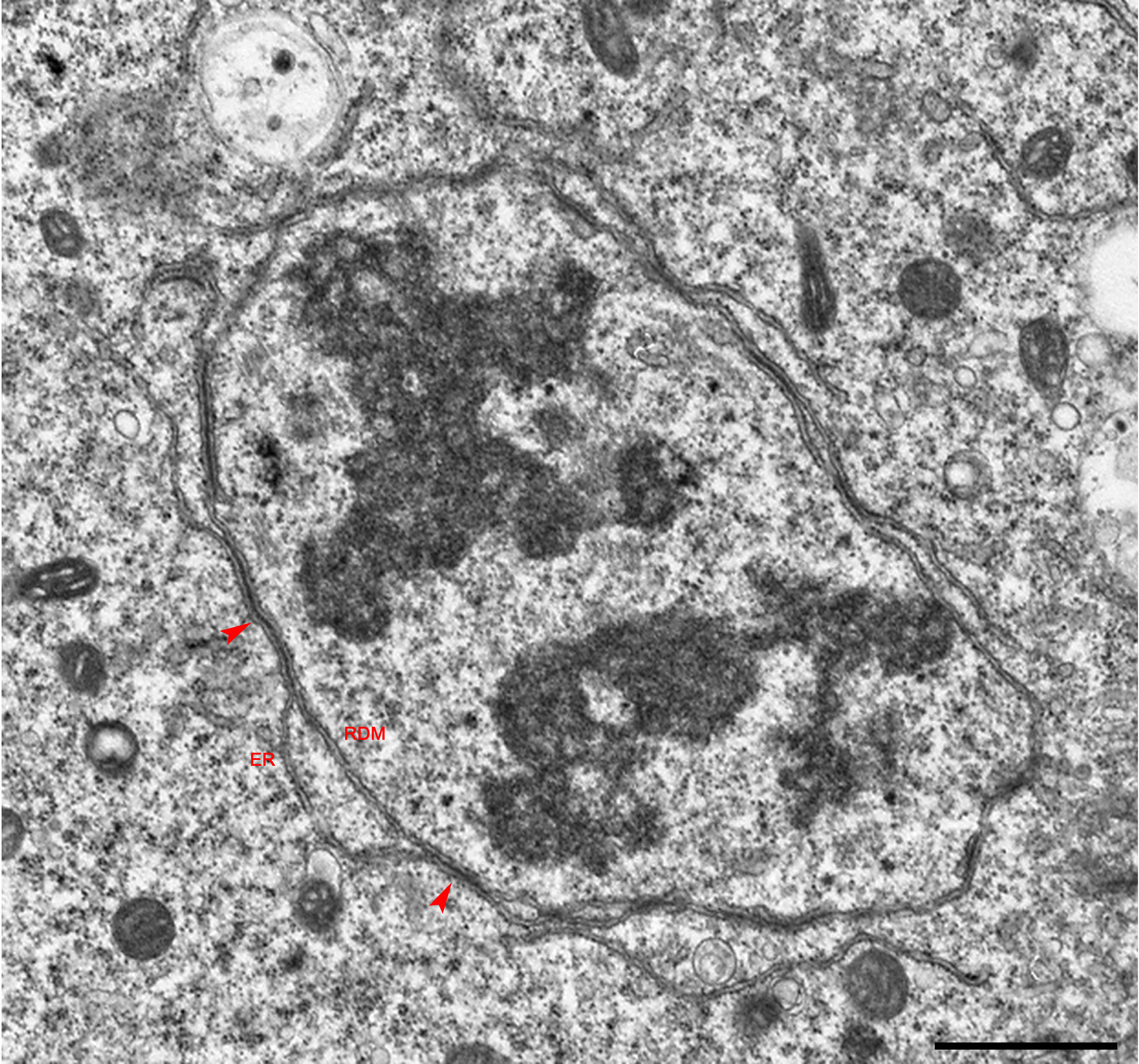

Supplement: Supplementary file 5 — Figure S5. The ER membranes occasionally become closely apposed to the nuclear envelope. The cell shown is in the PM3 stage, as its nuclear envelope comprises both regions of QNM and regions of RDM. The cell also contains ER membranes, which in some cases (arrowheads) become closely apposed to the nuclear envelope forming a structure that is apparently identical to a QNM. Scale bar: 1 μm. (TIF 12005 kb) [file 12915_2018_528_MOESM5_ESM.tif]

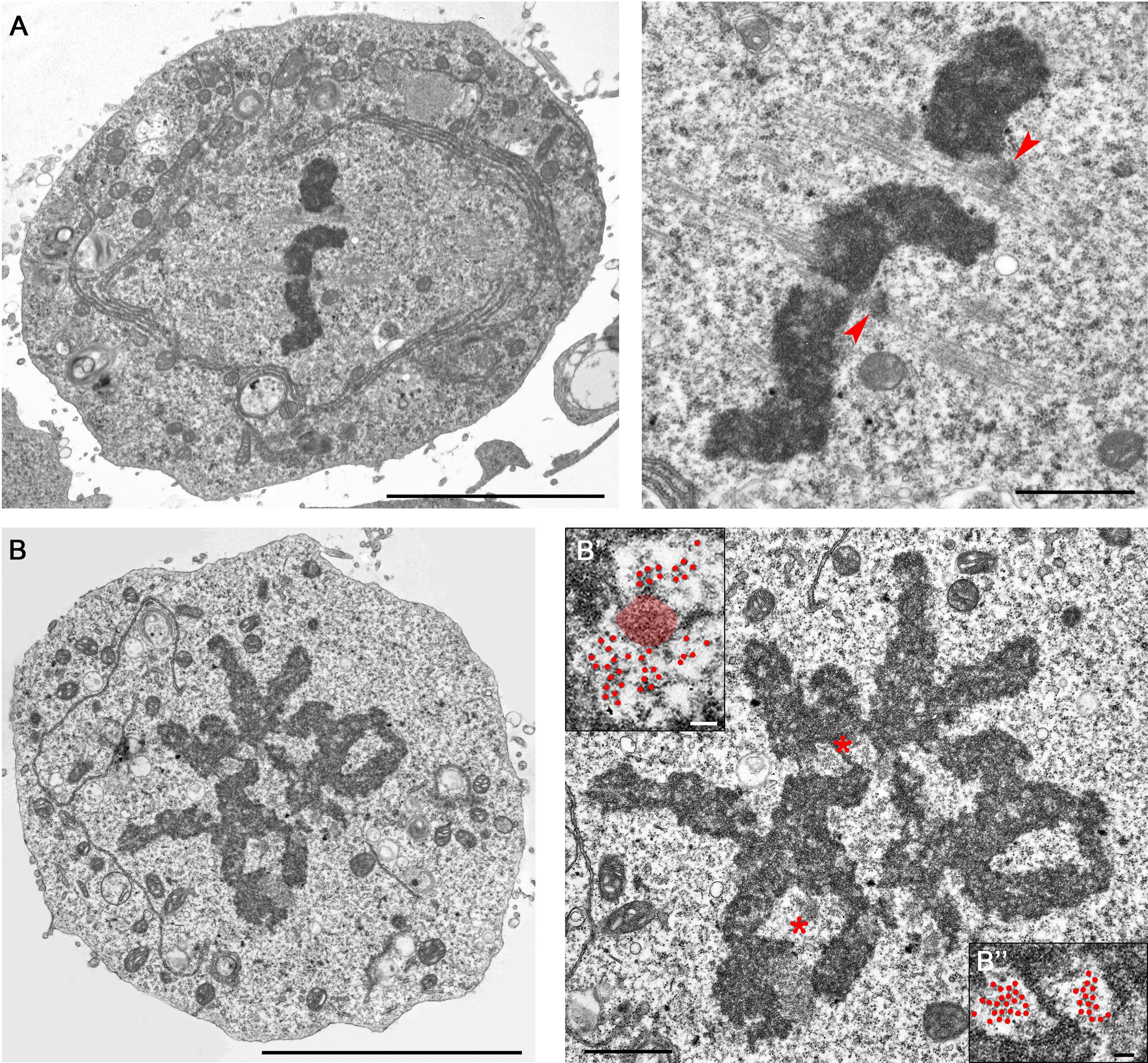

Supplement: Supplementary file 6 — Figure S6. Additional examples of metaphase S2 cells. a Longitudinal section showing stacks of parallel ER membranes and kinetochores with k-fibers (arrowheads in the magnified image). b Cross section through a metaphase plate shown at different magnifications. Asterisks in the right image indicate the regions magnified in the insets. The b' inset shows a kinetochore and associated MTs (both pseudo-colored in red). The b" inset shows two MT bundles that might be either k-fibers or interpolar MT bundles (see Fig. 9 and Additional file 12: Figure S12). Scale bars: left images, 5 μm; right images, 1 μm; insets, 0.1 μm. (TIF 18615 kb) [file 12915_2018_528_MOESM6_ESM.tif]

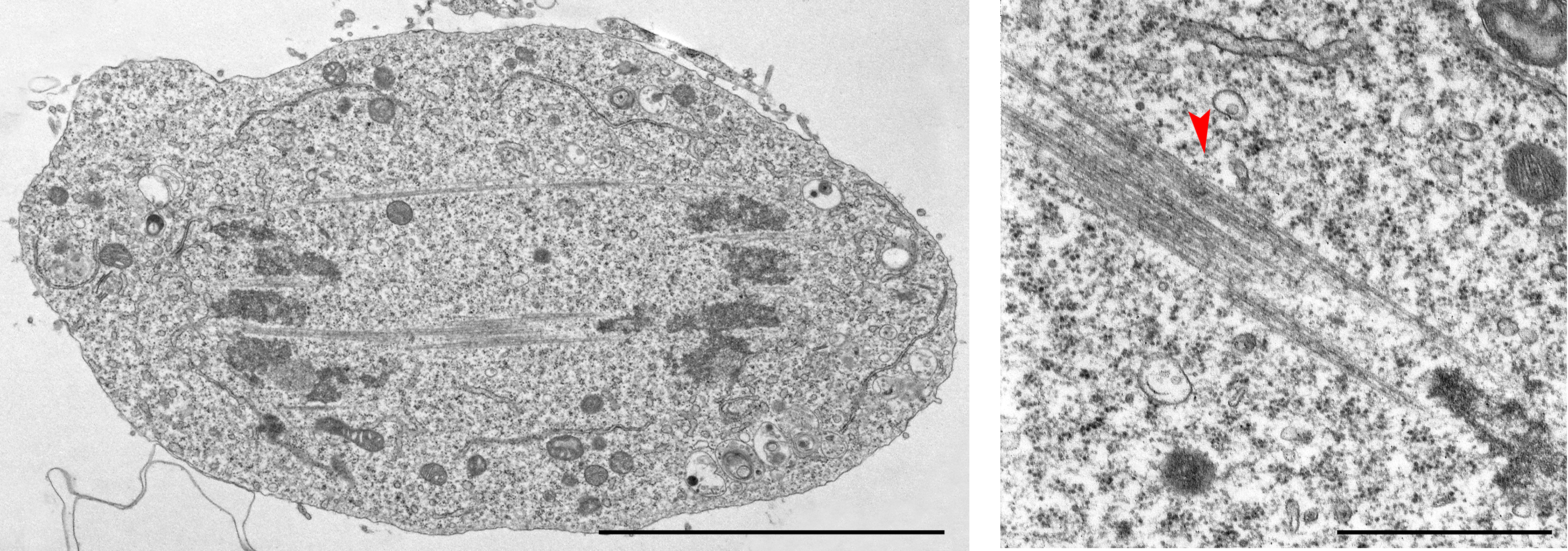

Supplement: Supplementary file 7 — Figure S7. An additional example of an S2 cell in late anaphase. Shown is the initial assembly of the central spindle. Note the apparently antiparallel MTs overlapping at the center of the cell (arrowhead in the magnified image on the right). Scale bars: left image, 5 μm; right image, 1 μm. (TIF 7426 kb) [file 12915_2018_528_MOESM7_ESM.tif]

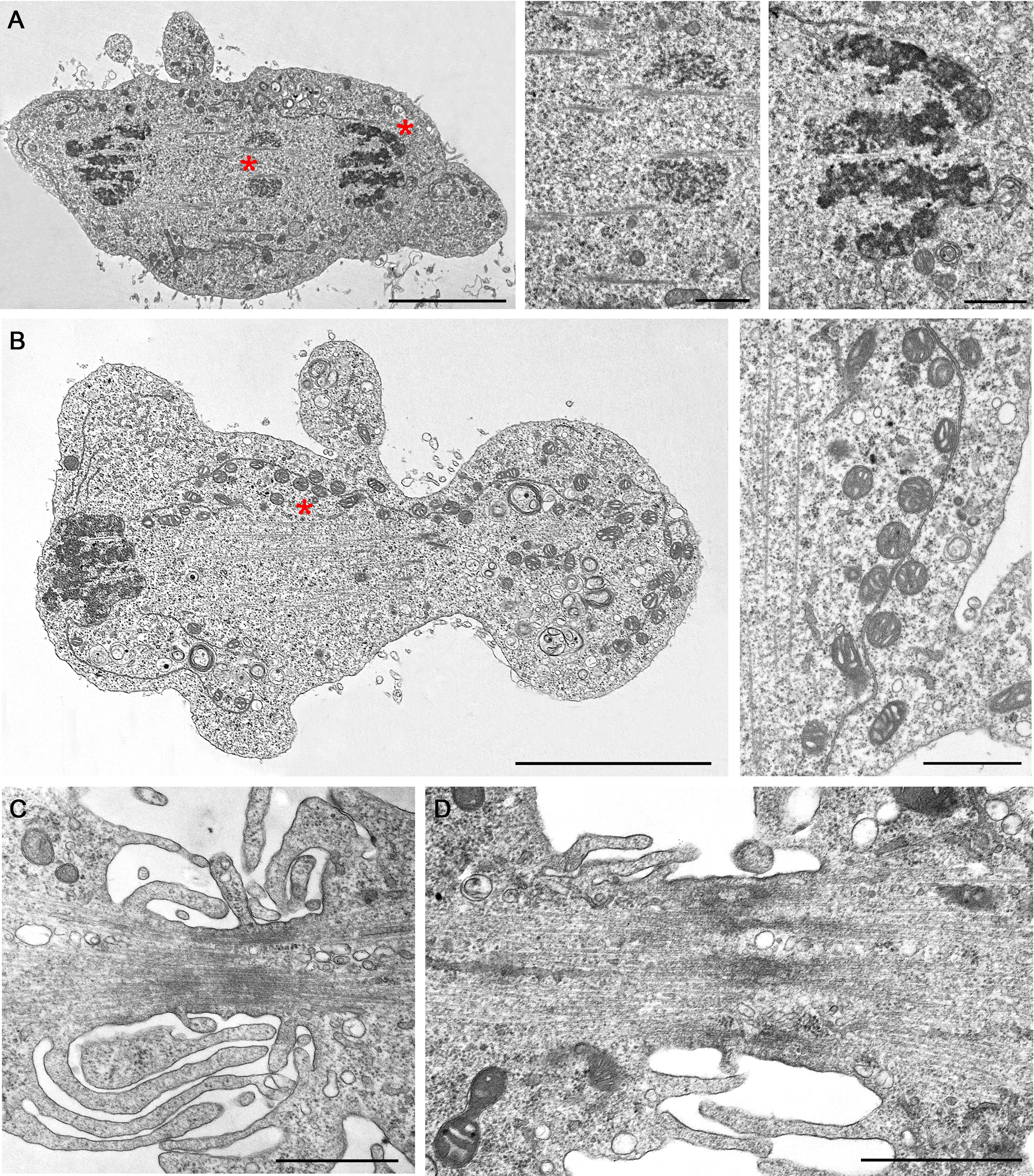

Supplement: Supplementary file 8 — Figure S8. Additional examples of telophase S2 cells. a Early telophase showing chromosomes partially surrounded by a double membrane, protrusions from the cell wall, and MT bundles at the center of the cell. b Early telophase with large protrusions from the cell wall and mitochondria attached to the ER membranes. a, b Asterisks indicate the cell regions shown at higher magnifications on the right. c, d Magnified images of late telophase intercellular bridges showing multiple membrane blebs and overlapping MTs in the middle of the bridges. Scale bars: 1 μm; except left images in A and B, 5 μm. (TIF 22942 kb) [file 12915_2018_528_MOESM8_ESM.tif]

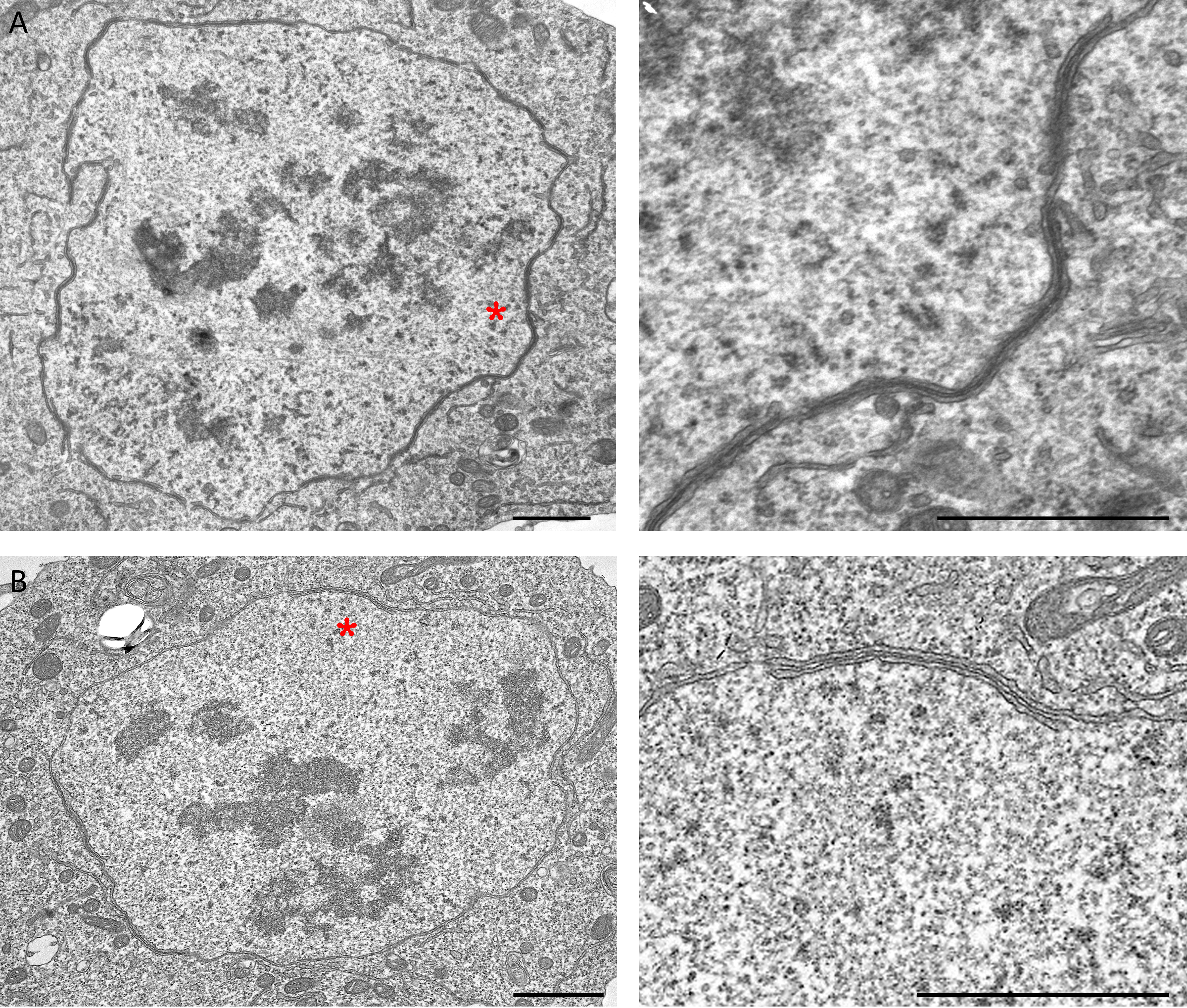

Supplement: Supplementary file 9 — Figure S9. The QNM forms in the absence of astral microtubules. a A prometaphase cell in which the nucleation of astral MTs is completely suppressed by RNAi-mediated depletion of the centrosome component Cnn. It exhibits a QNM comparable to that observed in cells in which aster formation is not inhibited. b A prometaphase-like cell from a culture treated for 3 h with colcemid shows patches of QNM. The asterisks indicate the cell regions shown at higher magnification on the right. Scale bars: 1 μm. (TIF 15859 kb) [file 12915_2018_528_MOESM9_ESM.tif]

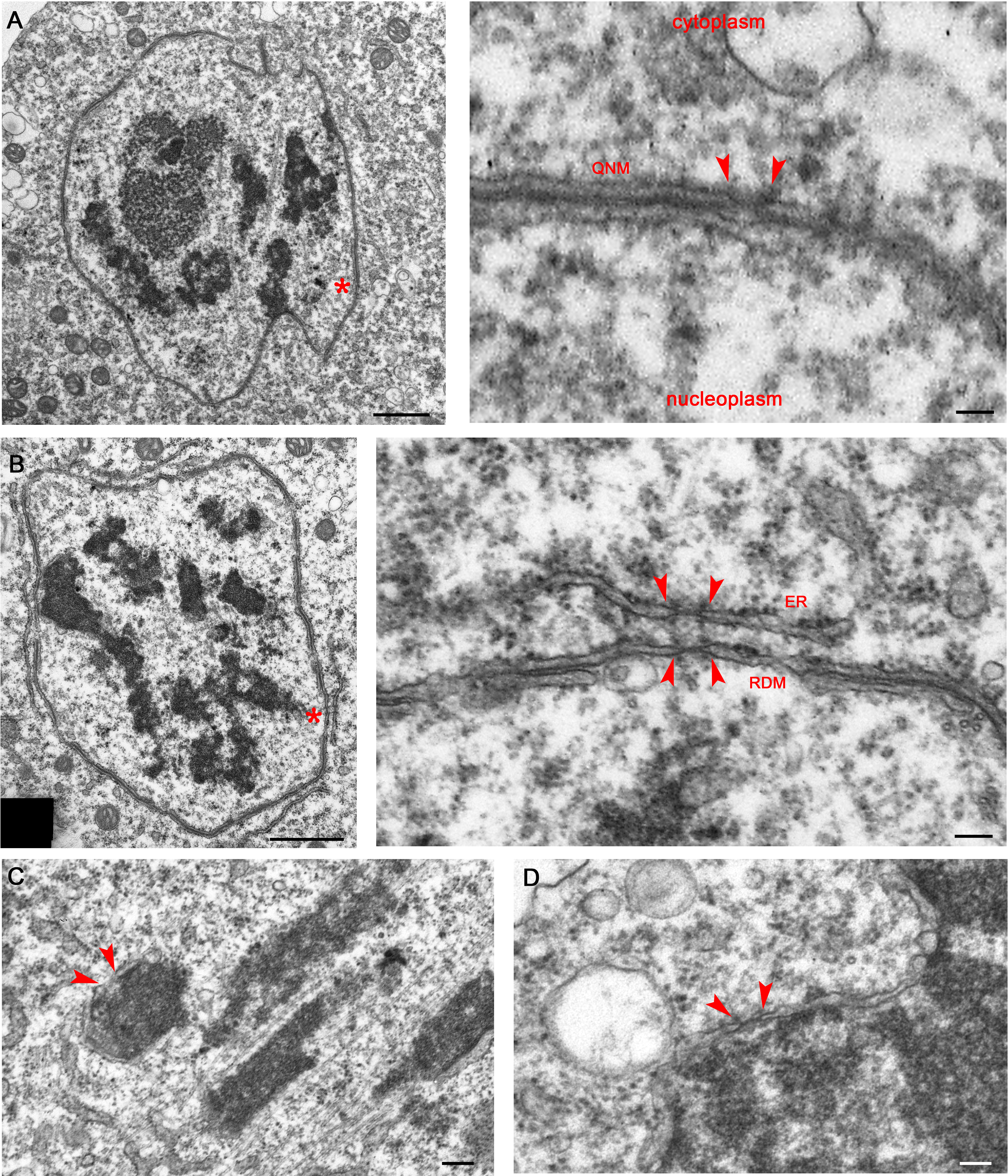

Supplement: Supplementary file 10 — Figure S10. Examples of ectopic nuclear pores in the outer component of the QNM and in the ER membranes. a PM3 cell showing an NPC in the outer component of the QNM (arrowheads in the magnified image shown on the right). b PM2 cell showing NPCs in the RDM and ER membrane approaching the nuclear envelope. a, b Asterisks indicate the cell regions shown at higher magnifications on the right. c, d Enlarged images showing NPC formation in the membranes surrounding c late anaphase and d telophase chromosomes. Scale bars: a, b left images, 1 μm; a, b right images, c, d, 0.1 μm. (TIF 19102 kb) [file 12915_2018_528_MOESM10_ESM.tif]

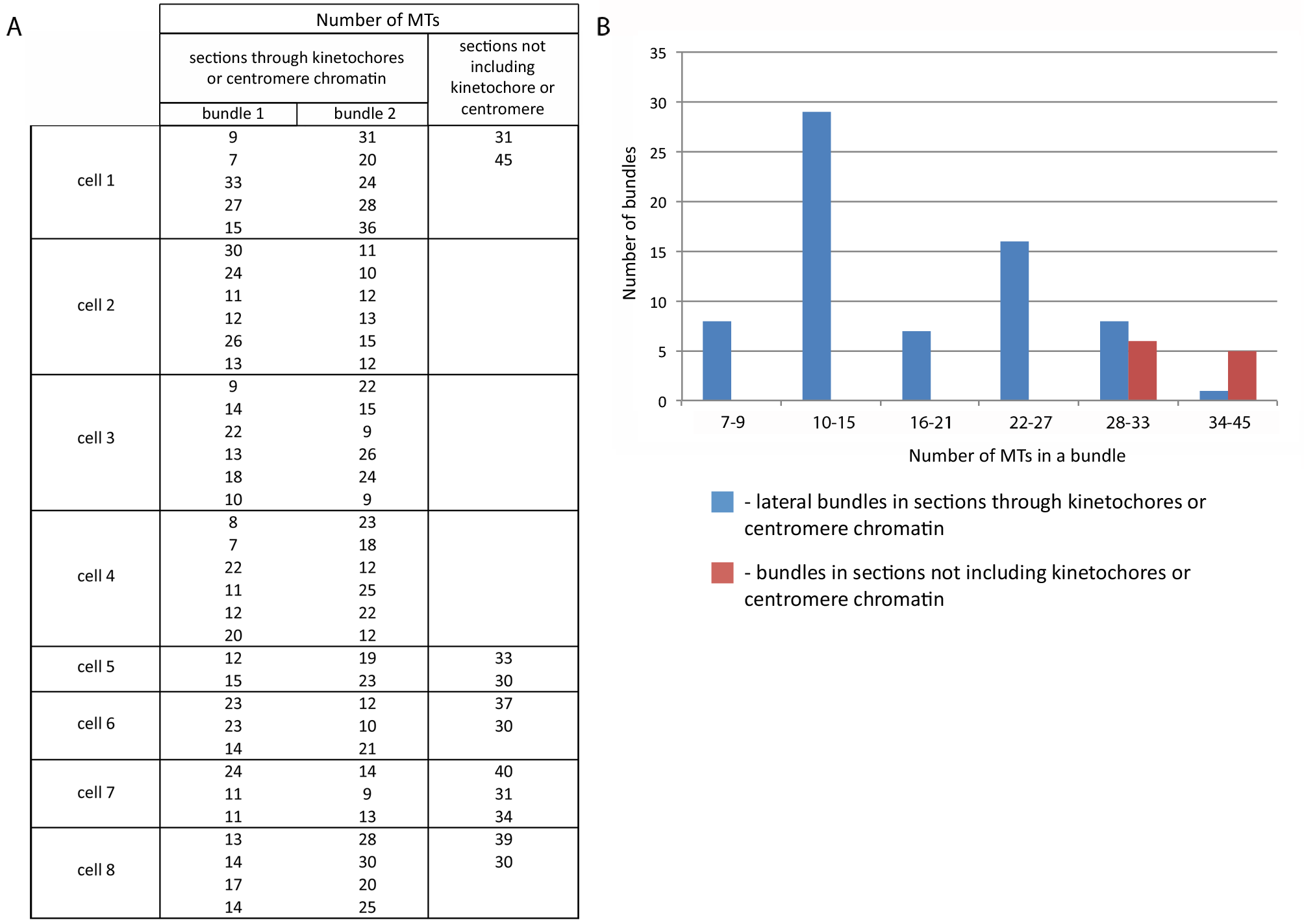

Supplement: Supplementary file 11 — Figure S11. Analysis of the lateral MT bundles in metaphase S2 cells. a MT bundles observed in a transverse section through the metaphase chromosomes of S2 cells. Only MT bundles within the metaphase plate were considered. Bundles outside this area, often containing four to nine MTs, were not taken into account. In sections through the kinetochore or the centromeric chromatin, we were always able to distinguish two separate MT bundles (indicated as bundle 1 and 2), which often contain different numbers of MTs. b Distribution of the MT bundles of different sizes (number of MTs) obtained by plotting the data shown in a. (TIF 216 kb) [file 12915_2018_528_MOESM11_ESM.tif]

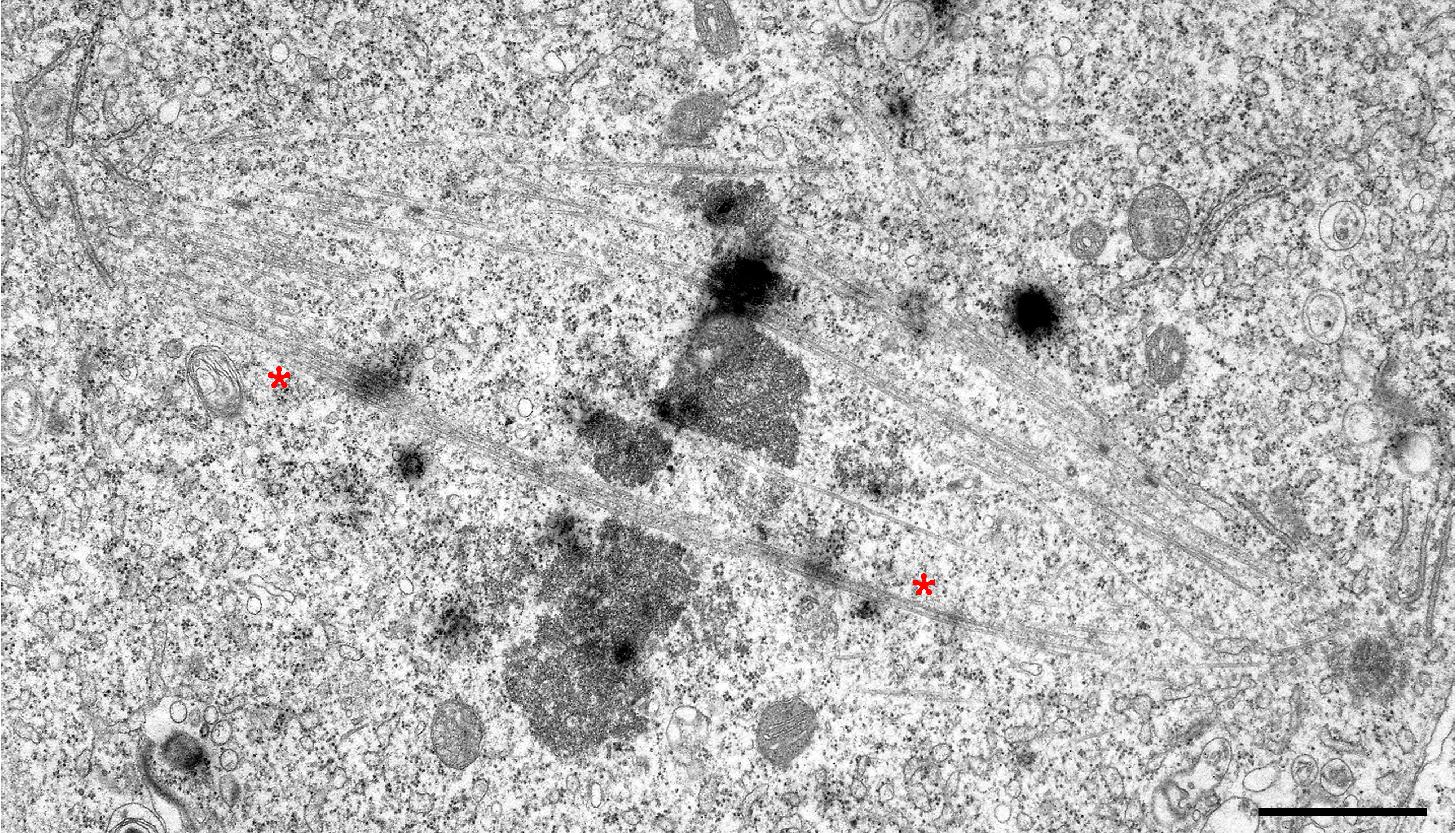

Supplement: Supplementary file 12 — Figure S12. Long MT bundles in a metaphase S2 cell. Shown is a long MT bundle running between the two spindle poles without interruptions (asterisks). Scale bar: 1 μm (TIF 12551 kb) [file 12915_2018_528_MOESM12_ESM.tif]
